# Modeling and Simulation of Mucus Flow in Human Bronchial Epithelial Cell Cultures – Part I: Idealized Axisymmetric Swirling Flow

**DOI:** 10.1371/journal.pcbi.1004872

**Published:** 2016-08-05

**Authors:** Paula A. Vasquez, Yuan Jin, Erik Palmer, David Hill, M. Gregory Forest

**Affiliations:** 1 Department of Mathematics, University of South Carolina, Columbia, South Carolina, United States of America; 2 Department of Mathematics, University of North Carolina at Chapel Hill, Chapel Hill, North Carolina, United States of America; 3 Marsico Lung Institute & Cystic Fibrosis Pulmonary Research and Treatment Center, University of North Carolina at Chapel Hill, Chapel Hill, North Carolina, United States of America; 4 Department of Physics and Astronomy, University of North Carolina at Chapel Hill, Chapel Hill, North Carolina, United States of America; 5 Department of Biomedical Engineering, University of North Carolina at Chapel Hill, Chapel Hill, North Carolina, United States of America; UNITED KINGDOM

## Abstract

A multi-mode nonlinear constitutive model for mucus is constructed directly from micro- and macro-rheology experimental data on cell culture mucus, and a numerical algorithm is developed for the culture geometry and idealized cilia driving conditions. This study investigates the roles that mucus rheology, wall effects, and HBE culture geometry play in the development of flow profiles and the shape of the air-mucus interface. Simulations show that viscoelasticity captures normal stress generation in shear leading to a peak in the air-mucus interface at the middle of the culture and a depression at the walls. Linear and nonlinear viscoelastic regimes can be observed in cultures by varying the hurricane radius and mean rotational velocity. The advection-diffusion of a drug concentration dropped at the surface of the mucus flow is simulated as a function of Peclet number.

## Author Summary

In the lungs, the airway surface liquid protects the airway epithelium from inhaled pathogens and particulates. It is well known that failure to properly clear mucus from the airways leads to chronic, even fatal, lung infections. To date, there is no validated constitutive model capable of recapitulating mucus rheology under diverse, physiological stress and deformation conditions. This gap has hindered studies into the causal relationship between mucus rheology and mucociliary clearance. Our modeling-experimental approach fulfills several purposes: to implement linear and nonlinear constitutive modeling of mucus from micro- and macro-rheology, to test constitutive modeling in an independent experimental system, to build a coarse-grained model of the PCL-mucus boundary condition, to measure and understand modifications in mucociliary transport during and after deposition of a controlled drug concentration, to measure and simulate both the flow and stress fields throughout the mucus layer, and to measure and simulate how the advection profiles in the culture couple with diffusion of particulates landing on the air-mucus interface. These results lay the groundwork for extension of the code to three dimensions and more realistic metachronal wave boundary conditions, both in cell cultures and in airways.

## Introduction

The propulsion of mucus in human airways to the trachea by the collective, coordinated action of cilia, known as mucociliary clearance (MCC), remains an outstanding modeling and computational challenge. In the lungs, the airway surface liquid (ASL) protects the airway epithelium from inhaled pathogens and particulates. It is well known that failure to properly clear mucus from the airways leads to chronic, even fatal, lung infections. Consequently, a MCC model that allows for quantitative predictions of ASL transport as a function of mucus rheological properties is valuable for detecting compromised MCC and assessing mucolytic-based treatments.

The ASL is mainly composed of two concentric, annular layers: the periciliary liquid (PCL) layer bordering the epithelium that surrounds the cilia, forming an active medium with apparent polymer brush-like properties [1]; and, a mucus layer between the PCL and air in the core of airways. Mucus is an extremely complex, viscoelastic gel-like fluid, whose properties vary dramatically with disease and disease progression. To date, there is no validated constitutive model capable of recapitulating mucus rheology under diverse, physiological stress and deformation conditions. This gap has hindered studies into the causal relationship between mucus rheology and mucociliary clearance. Nonetheless, important advances have been made in understanding the different physiological, biochemical, and mechanical processes at play in MCC.

In general, one can divide the modeling of MCC into three major components: formulation of a constitutive model for mucus based on rheological data, understanding the mechanism (forcing condition) for mucus propulsion either by an individual cilium or carpets of cilia, and development of numerical methods to solve the resulting system of equations [2, 3]. Validation of each component in airway simulations is essentially impossible due to the lack of *in vivo* experimental data and resolution of airway mucus transport.

**Fig 1:**
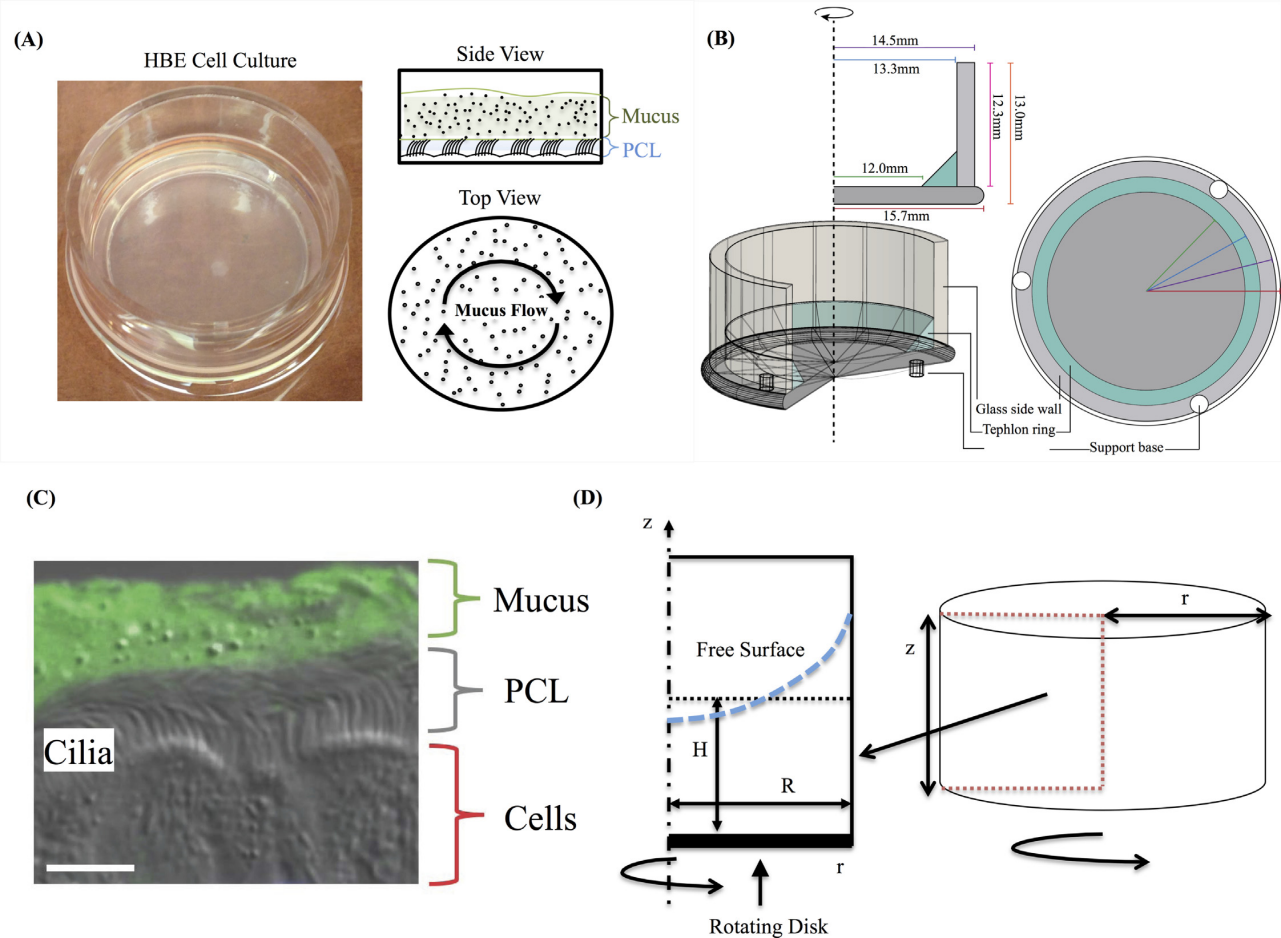
System’s description. **(A)** Schematic representation of a Human Bronchial Epithelial (HBE) cell culture. **(B)** HBE cell culture geometry. **(C)** combination DIC with fluorescent labeled mucus of mouse airway section. Scale bar 7 um. Image courtesy of Camille Ehre, UNC Marsico Lung Institute. **(D)** HBE cell culture in cylindrical coordinates and reduction from 3D cylinder to 2D rectangular region based on the axisymmetric assumption.

**Fig 2:**
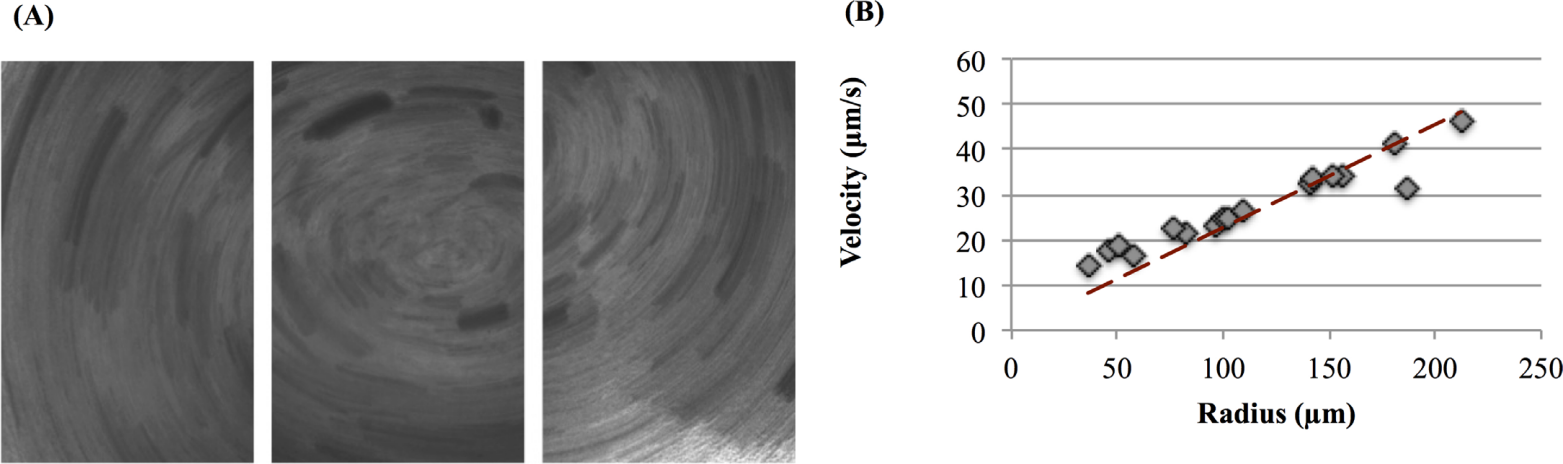
Demonstration of rotational mucus transport in HBE cell cultures. **(A)** Traces of 1 mm fluorescent micro-spheres at the culture surface from a 5 second time lapse exposure. **(B)** Linear velocities of the particles versus distance from the center of rotation. Data extrapolated from [25].

A transformative *model system* for the study of mucociliary clearance is provided by human bronchial epithelial (HBE) cell cultures. These cultures are grown from donor cells and at maturity consist of epithelial tissue covering a porous membrane with a nutrient solution below the membrane. The membrane allows passage of nutrients and buffer fluid to sustain the PCL and mucus layers. The complete system consists of epithelial cells, a layer of fluid surrounding the cilia carpet (PCL), and an overlying mucus layer (see [4] and Fig 1). A great deal of studies have been performed using these cylindrical cultures [5–14] and their mean macroscopic rotational flow has been quantified via microscopy (see [14] and Fig 2). A variety of modeling efforts have focused on understanding cilia-driven viscous and viscoelastic fluid transport [13–20], and in modeling of mucus flow in cell cultures in particular [18–20]. The objective of the present study is to likewise use computational modeling to predict mucus transport within HBE cultures, but with constitutive equations and model parameters derived from linear and nonlinear experimental data. We are interested in the roles that mucus rheology, wall effects, and HBE culture geometry play in the development of flow profiles and the shape of the air-mucus interface.

In recent years, the formulation of new mathematical algorithms together with an increase in computational power have made it possible to explicitly model the interaction between beating cilia and mucus [15–19]. In this type of modeling, the cilia are given internal structure and the fluid-structure interactions are explicitly formulated and solved. Several studies have shed light on the intricate relationship between cilia beating and mucus transport [17–22]. These numerical models employ either Newtonian fluid assumptions or canonical non-Newtonian constitutive equations to model the mucus layer. Within this class of studies, we note in particular two multiscale models developed by Mitran [17, 23]. In one model [17], internal motor forces within each cilium adapt to local flow conditions guided by a minimization of work hypothesis, leading to cilia synchronization and metachronal wave formation. This computational platform was implemented in a planar geometry, with a single-mode, upper convected Maxwell constitutive equation for mucus and a viscous fluid surrounding cilia; among other features, the simulations show cilia tips penetrating the mucus layer at full extension in the power stroke.

Smith and co-workers [20–22] developed a series of multilayer models to specifically model cell cultures. The ASL consists of a viscoelastic mucus layer, bordered below by a thin traction layer to mimic cilia-mucus engagement and propulsion. The traction layer is bordered below by a periciliary liquid layer that is treated as a Newtonian fluid moving through a porous medium. In this work, a linear viscoelastic constitutive equation, namely the Maxwell model, was used to capture the elastic and viscous effects for both the traction and mucus layer. Within their conclusions, the authors state that the “ciliary beat frequency was a crucial determinant of efficient transport, and ‘watery’ mucus was transported more efficiently than normal mucus.” However, other viscoelastic effects could not be discerned as the Maxwell model only predicts linear viscoelastic behavior. Sedaghat *et al*. [18] used a hybrid finite difference-lattice Boltzmann method to study the transport of mucus and PCL. Their work employed an Oldroyd-B constitutive model and concluded that both mucus viscosity and relaxation time control MCC transport processes. Lee *et al*. [19] used an immersed boundary method to test the effects of mucus viscosity, surface tension, cilia beat frequency, number of cilia, and thickness of the PCL layer. They conclude that the last three factors critically affect the efficiency of MCC.

As mentioned above, there exists a wealth of MCC modeling approaches that focus on the impact of cilia beating, with the above work giving a brief sampling of the literature. We point the reader to these references and to the rigorous review on modeling mucociliary motion by Smith *et al*. [22]. In the work presented here, lessons learned from these studies will be applied to coarse-grain the driving conditions imposed by the cilia, and to focus more on a faithful nonlinear rheological characterization of mucus from experiments on HBE cultured mucus. We coarse-grain the cilia-mucus propulsion mechanism as follows. First, the thickness of the mucus layer is considered to be much smaller than the metachronal wavelength, so that the cylindrical flow can be assumed axisymmetric, i.e., there is no dependence in the angular direction. Second, the effects of individual cilia and cilia patches are averaged to impart a macroscopic oscillatory boundary condition on the velocity at the base of the mucus layer. The aim is to explore the qualitative flow and air-mucus interfacial features of HBE cell cultures due to experimentally derived HBE mucus rheological constitutive modeling, with an admittedly coarse model of cilia-mucus forcing. For this reason, quantitative comparisons with cell culture flow and interfaces are not possible. We either have to generalize our models to include active cilia in a periciliary liquid layer, or we could design a culture experiment with cilia replaced by a lower plate with the controlled forcing motion given below.

Another study by Mitran [23] builds a computational platform based on concepts from polymer gel theory, including a distribution of microscopic interactions that up-regulate the rheological properties of mucus. This platform and microscopic dynamics have not been validated with experimental data so the predictions are meant to establish proof-of-principle of the numerical tools. One of the gaps our study proposes to fill is to model mucus with viscoelastic constitutive equations that capture nonlinear viscoelastic behavior such as shear thinning vs. thickening and stress softening vs. hardening, and that resolve multiple timescales of stress relaxation. We do not resolve the microscopic processes for this paper, e.g., entanglement dynamics and molecular crosslinking kinetics, since we have no experimental basis for any of these processes in mucus. From this multi-mode constitutive model basis, we fit constitutive parameters directly from linear and nonlinear experimental data on HBE culture mucus. In addition, although the role of stress relaxation time has been studied within a mucus transport framework, we explore multiple relaxation modes that collectively can capture the rheological data whereas single modes fail miserably.

We use human bronchial epithelial cell cultures as an *ex vivo* model system to develop and validate a computational model of mucus transport. These cultures, developed by Lechner and coworkers [24], grow to confluence with cell differentiation into goblet cells that produce mucin proteins and ciliated cells that sprout active cilia, forming epithelial tissue that draws nutrients and water from the reservoir below the membrane. Within weeks, an air-mucus interface forms over a mucus layer of ~ 10–50 microns, with a PCL between mucus and tissue approximately 7 microns thick (Fig 1). Ciliated cells (~200 cilia per cell, each ~ 8 microns long at full extension of the power stroke) coordinate their individual power-return stroke with ~ 10–15 Hz frequency, propelling the mucus layer in the culture either clockwise or counterclockwise. The higher rotational velocities near the center create flow that resembles a hurricane from above, and thus this pattern has come to be named a “mucus hurricane” (Fig 2A). Once coordinated transport of the mucus layer is established, fluorescent tracer particles [4, 14], gold nanorods [25], or endogenous cellular debris [25, 26] can be tracked to gain flow profile information. For cultures whose cilia synchronize and transport mucus around the dish, a true *ex vivo* model assay is provided for a detailed study of a wide range of pulmonary physiology. Cultures can be obtained from donors of various backgrounds, including those that carry genetic diseases such as Cystic Fibrosis (CF) [27–32], Primary Cilia Dyskinesia (PCD) [33, 34], and asthma [35, 36] that accurately mimic pathological phenotypes. Further, cultures can be treated with inflammatory molecular, pathogenic material, and other environmental factors such as wood and cigarette smoke to further mimic pathological phenotypes ranging from the common cold [37] to Chronic Obstructive Pulmonary Disease (COPD) [38–41] and serve as a testing platform for underlying pathology causes and potential correctors.

To construct a nonlinear constitutive model of mucus we use experimental data from micro- and macro-rheology. For additional information, we refer to recent studies of biochemical composition [42], macro-rheological [4], and microrheological [43] properties of sputum and culture mucus. As discussed above, for this initial study, we avoid the open question of cilia-mucus propulsion, and idealize the forcing mechanism of the power and return stroke as though the cilia were uniformly synchronized. The actual momentum transfer mechanism between the PCL and mucus layers remains one of the most important unsolved problems in lung mechanics. We do not address this problem in the current paper. Instead, we draw from previous work [21, 23] and treat the PCL as a moving solid boundary, averaging out the scales of the coordinated cilia to study the mean mucus transport features due to a homogeneous dynamic cilia carpet. This idealization affords an axisymmetric reduction to two space dimensions (Fig 1D).

Next, we develop a computational simulation of axisymmetric mucus transport in a cell culture, including both transient and stationary flows. Our simulations illustrate remarkable qualitative differences in the flow and air-mucus interface due to the nonlinear rheology of mucus versus a viscous fluid assumption. To model the mucus layer we employ a five-mode Giesekus nonlinear viscoelastic constitutive law. The Giesekus model is a canonical model that captures shear thinning and first and second normal stress generation in shear [44]. The number of modes and their parameters are chosen for their ability to mimic HBE mucus rheology. The novelty of this approach therefore lies in the accuracy of the five-mode Giesekus model in approximating the experimentally verified relaxation spectrum of mucus. We solve the full system of governing equations for transient and stationary axisymmetric flow fields and the air-mucus interface, and explore the mass transport of mucus as a function of the Giesekus constitutive properties and the imposed driving condition at the lower plate.

Finally, we apply the time-dependent flow in the culture geometry to simulate advection-diffusion of small molecules deposited at the air-mucus interface in order to investigate how mucus flow affects drug delivery to the underlying tissue in cell cultures. A similar study was performed by Smith *et al*. [21] motivated by experiments of Matsui *et al*. [14]. In Smith *et al* work it was found that the fluid transport in HBE the cultures was greater than the one predicted by their traction layer model. The authors conjectured that this was due to vertical mixing near the interface. In this work, we use the Peclet number, a dimensionless ratio of advective to diffusive transport, to draw conclusions about the transport of diffusing particles in a cell culture. In this context, the cell cultures afford an experimental system with a tunable Peclet number, which can be varied by modifying either the viscoelasticity of the mucus layer, the driving conditions for mucus transport, or the radius of the portion of the cell culture that exhibits the hurricane behavior. In particular, different concentrations of solids (mucin molecules, proteins, and salts) in mucus have been shown to dramatically alter viscoelastic properties and diffusive mobility of 200 nm and larger probe particles [43] in HBE mucus.

The objective of this work is therefore twofold. First, we present a model capable of reproducing nonlinear flow behavior consistent with experimental characterization of mucus rheology from mucus harvested from the very HBE cell cultures we are modeling. Then, in a qualitative comparison with experimental observation, we use the model for several purposes: to explore the implications of nonlinear rheology of mucus, e.g., the degree of normal stress generation and its impact on the air-mucus interface; to explore the generation of higher harmonics in flow and stress components from a single frequency of forcing and the spatial extent of these nonlinearities; and finally, to explore advection-diffusion of chemical concentrations deposited at the air-mucus interface. Because we coarse-grain the cilia driving condition to a rotating lower plate and axisymmetry, the only sensible comparisons of the interface and flow field are qualitative. These developments contribute along with the contributions noted above toward a foundation for resolution of cilia-mucus forcing conditions, microscopic processes in both cilia forcing and mucus nonlinearity, mucociliary clearance in human airways, all in fully three-dimensional simulations.

## Methods

As noted above, for this initial study we suppress heterogeneity of the cilia forcing condition, tantamount to a two-dimensional long-wave limit of the ciliary metachronal wave. A flat rigid disk that moves with a prescribed, non-monotonic, angular velocity, which mimics the power and return strokes of cilia, thereby replaces the ciliated carpet. This treatment of the cilia carpet is self-consistent with axisymmetry of the three flow variables, pressure, and free surface, affording a reduction in the computation domain from three (*r*, *θ*, *z*) to two (*r*, *z*) space dimensions, as shown in Fig 1D. Briefly, the computational domain consists of a cylinder of radius *R* and height *Z*, and a layer of viscoelastic fluid of initial uniform height *H*. The fluid is set into motion by a rotating disk at the bottom of the cylinder with an angular velocity, *ω*, which we vary to mimic forward and return strokes of cilia. In addition, the air-liquid interface is free, with surface tension estimated from the literature [45–48], as explained below.

### Governing Equations

For an incompressible, isothermal, non-Newtonian fluid, the conservation of mass and momentum equations are,

∇⋅u=0, (1)

$$\rho \left(\frac{\partial u}{\partial t}+u\cdot \nabla u\right)=-\nabla p+\eta_{s}\Delta u+\nabla \cdot \tau +\rho g.$$ (2)

Where *ρ* is the fluid density and *η*_*s*_ is the solvent viscosity. To close the system, a constitutive equation for the extra stress tensor, ***τ***, is needed. We use both linear and nonlinear viscoelastic data from cell culture mucus to construct the constitutive model, as follows: we assume a multi-mode nonlinear viscoelastic model,

$$\tau =\sum \tau_{i},$$ (3)

where each mode is governed by a constitutive equation of the so-called Giesekus form,

$$\tau_{i}+\lambda_{i}\tau_{i(1)}+\frac{\alpha_{i}\lambda_{i}}{\eta_{p,i}}\tau_{i}\cdot \tau_{i}=\eta_{p,i}\dot{\gamma }$$ (4)

Here, for each mode, *λ*_*i*_ is its relaxation time and *η*_*p*,*i*_ is its polymer contribution to the viscosity. The total viscosity, *η*_0_, is the sum of the solvent and mode contributions, *η*_0_ = *η*_*s*_ + ∑*η*_*p*,*i*_. In addition, $\dot{\gamma }=(\nabla u+\nabla u^{T})$ is the rate of strain tensor, and the subscript notation ***τ***_(1)_ denotes the upper convected derivative (that guarantees the model is invariant under rigid body motions or coordinate changes), given by

$$\tau_{\left(1\right)}=\frac{\partial \tau }{\partial t}+u∙\nabla \tau -\tau ∙\nabla u-\nabla u^{T}∙\tau .$$

In the Giesekus model, *α* is a tunable nonlinearity parameter, called the mobility parameter; in the limit *α* = 0, the Giesekus model reduces to the Upper Convected Maxwell (UCM) model. The UCM model captures the fundamental viscoelastic property of normal stress generation in shear flows, *whereas the quadratic nonlinearity of the mobility term in the Giesekus model is necessary to capture shear thinning*.

### Rheological Characterization of Mucus

**Fig 3:**
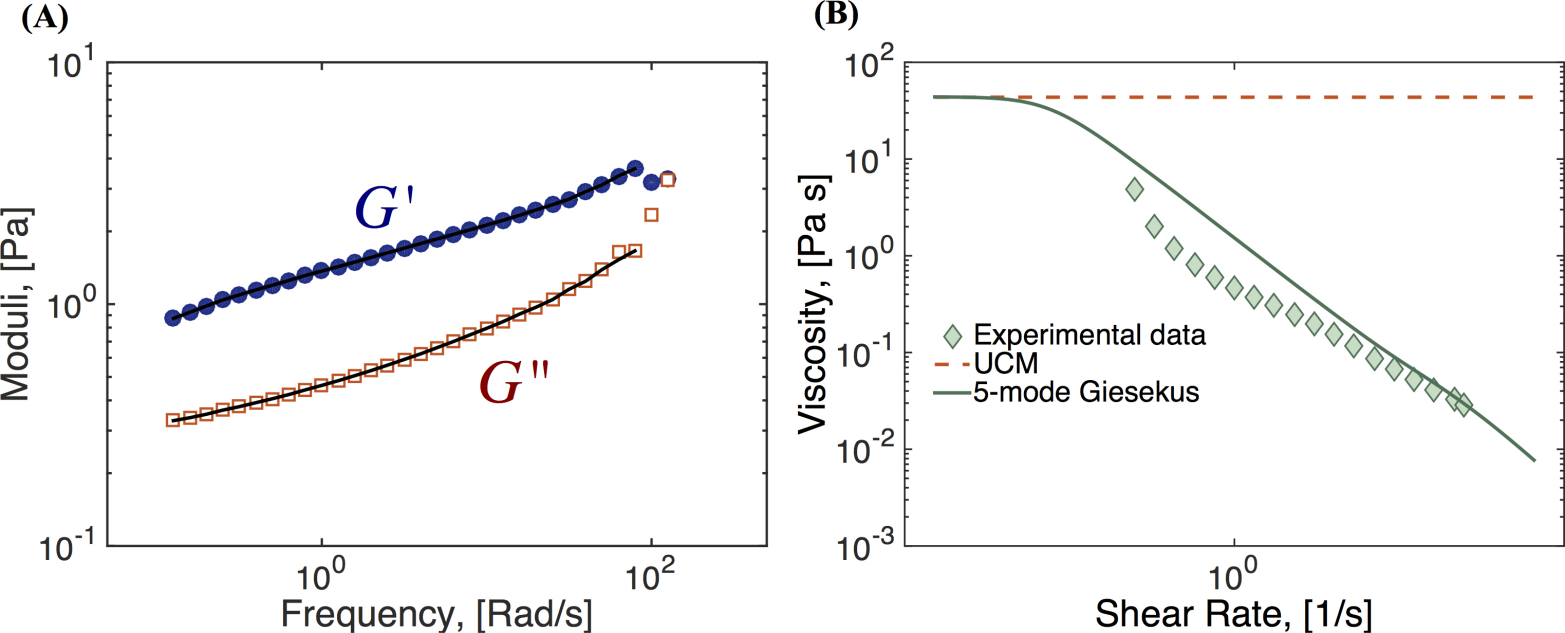
Fluid characterization. **(A)** Experimental data of storage (*G*’) and loss (*G*”) moduli for 2.5 wt% HBE mucus across a frequency range (symbols), together with the corresponding fit to 5 UCM modes (lines). **(B)** Viscosity vs. shear rate data (symbols) for 2.5 wt% HBE mucus and corresponding fit to a sum of 5 Giesekus modes (line). The curve shown is the best fit under the condition 0 ≤ *α* ≤ 0.5. Model parameters are given in Table 1. Data courtesy of Jeremy Cribb and David Hill, UNC-CH.

To select the modeling parameters for the multi-mode Giesekus model, our approach is to use experimental data to identify linear (small amplitude) storage and loss moduli across a physiological frequency range, and then to use a discrete set of UCM modes (*α* = 0) that give a fit to the experimental data. We use data generated from a fixed-strain frequency sweep in a cone and plate TA Instruments AR-G2 rheometer and a 2.5 wt% (weight percent) HBE mucus sample, representative of the healthy range of human lung mucus. The data and corresponding fit using five UCM modes is shown in Fig 3A, with the parameter results shown in Table 1. The shear thinning of mucus is captured by the mobility parameter *α*, which we restrict to the range 0 ≤ *α* ≤ 0.5, since values greater than 0.5 result in non-monotonic flow curves. To find the best fit for the nonlinear mobility parameters per Giesekus mode, we use rheometric data of the shear-thinning flow curve (the viscosity versus shear rate), as shown in Fig 3B. In summary, relaxation times and contributions to the total viscosity are used to find the best fittings to the data in Fig 3A, while Fig 3B was used to find the values of the parameter alpha for each mode.

**Table 1:** Parameters for five-mode Giesekus model.

Mode	Relaxation time, [s]	Modulus, [Pa]	Giesekus parameter, *α*
1	0.0089	3.3472	0.2
2	0.0821	0.7551	0.3
3	0.4660	0.5350	0.5
4	3.1290	0.4543	0.5
5	49.733 (*λ*_*L*_)	0.8486	0.5

### Boundary Conditions

No-slip flow boundary conditions are imposed at the solid walls of the cylinder, along the central axis of the cylinder, *r* = 0, we impose a vanishing Neumann condition in the radial coordinate on all flow variables. These conditions are summarized in Table 2. To reconcile the fact that the azimuthal velocity is zero at the vertical walls and non-zero at the bottom wall, we use a boundary layer of the form,

$$u_{\theta }(r,t)=U(r,t)\left(1-\exp\left[-\frac{R^{2}-r^{2}}{\epsilon \;R^{2}}\right]\right),$$

where *U*(*r*,*t*) is the imposed velocity at the bottom plate and *ϵ* = 0.05 in our simulations.

**Table 2:** Imposed boundary conditions.

Variable	Condition at *z* = 0	Condition at *r* = *R*	Condition at *r* = 0
	(Bottom plate)	(Cylinder wall)	(Center of the culture)
*u*_*r*_	*u*_*r*_ = 0	*u*_*r*_ = 0	$\frac{\partial u_{r}}{\partial r}=0$
*u*_*z*_	*u*_*z*_ = 0	*u*_*z*_ = 0	$\frac{\partial u_{z}}{\partial r}=0$
*u*_*θ*_	*Imposed velocity (for example Eq 5)	*u*_*θ*_ = 0	$\frac{\partial u_{\theta }}{\partial r}=0$
***τ***	***τ*** = 0	***τ*** = 0	$\frac{\partial \tau }{\partial r}=0$

*The contradicting conditions for the azimuthal velocity are resolved using a boundary layer as explained in the text.

The beating of a single cilium is periodic and asymmetric; consisting of two phases: a forward power stroke (or effective stroke) in which the tips (~ 1 micron) of the fully extended cilia penetrate the mucus layer, and a return stroke (or recovery stroke) in which the cilia do not engage the mucus layer but still induce some mild backflow [25]. The cilia beat in a coordinated fashion over several cell diameters, creating metachronal waves that propel the mucus above. The rotational movement of the mucus induced by the cilia carpet is locally periodic. According to our experimental observations, mucus moves in the angular direction about 10 μm in one forward cycle of 0.1s and then moves about 5 μm in one backward cycle of 0.1s, measured near the edge of the cell culture (Adopted from data collected in [25]). We coarse-grain the cilia propulsion mechanism into an imposed dynamic velocity condition *u*_*θ*_|_*z* = 0_ on the flat base of a cylinder. As mentioned before, the rotational velocity within the mucus hurricane is approximately linear with respect to the radial distance from the center. Therefore, as a first step, we model the cilia driving condition with an angular velocity ω, where *u*_*θ*_|_*z* = 0_ = *ωr*.

#### Swirling flow

This type of flow is obtained by using a constant angular velocity ω = *ω*_0_. Note that, this does not match the periodic and asymmetric driving force of the cilia carpet. However, swirling flow provides a simple case where we can validate our numerical method, as well as compare the difference between Newtonian fluids and nonlinear viscoelastic fluids.

#### Cilia-driven flow

In order to simulate the two-phase (power, return) periodic cilia stroke, we use a periodic angular velocity, *ω* = *f*(*t*), where *f*(*t*) is a piece-wise sinusoidal function given by

$$f\left(t\right)=\{\begin{array}{l} \;P_{0}\;\sin\left(\omega_{p}\;t\right),\;\mathrm{for}\;0<t<\frac{\pi }{\omega_{p}},\\ \;R_{0}\;\sin\left(\omega_{r}\;t\right),\;\mathrm{for}\;\frac{\pi }{\omega_{p}}<t<\frac{\pi }{\omega_{p}}+\frac{\pi }{\omega_{r}}, \end{array}$$ (5)

where *P*_0_ and *R*_0_ are the angular velocity amplitudes during the power and return strokes, respectively. Similarly, *ω*_*p*_ and *ω*_*r*_ are the frequencies of the power and return strokes. Based on our experimental observation, we select *P*_0_, *R*_0_, *ω*_*p*_, and *ω*_*r*_ by taking into account the experimentally observed duration of one cycle,

$$\frac{\pi }{\omega_{p}}=0.1\;s,\;\frac{\pi }{\omega_{r}}=0.1\;s,$$

and the observed movement of the cilia,

$$\int_{0}^{0.1}P_{0}\;r\;\sin\left(\omega_{p}\;t\right)dt=1\times 10^{-5}\;m,\;\int_{0.1}^{0.2}{\;R}_{0}r\;\sin\left(\omega_{r}\;t\right)dt=5\times 10^{-6}\;m.$$

**Fig 4:**
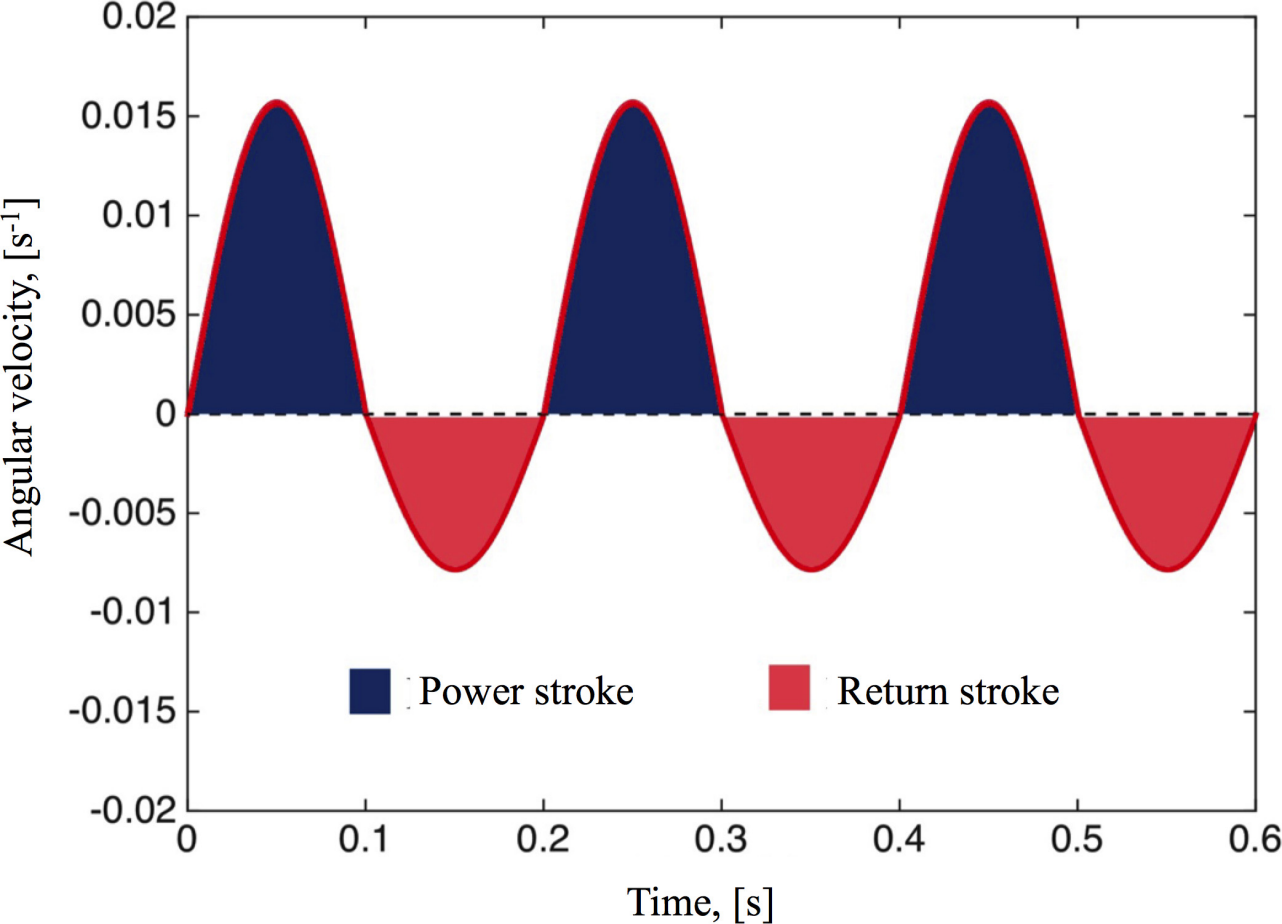
Imposed boundary condition. The oscillatory driving condition *f*(*t*) imposed at the bottom plate to mimic cilia power and return strokes and given by Eq (5).

With *r* = 10mm, we have *P*_0_ = 1.57 × 10^−2^ Rad/s, *R*_0_ = 7.9 × 10^−3^ Rad/s, *ω*_*p*_ = 10*π* Rad/s and *ω*_*r*_ = 10*π* Rad/s. A typical driving condition is shown in Fig 4 where the power and return strokes’ portions of the velocity profile are highlighted.

### Free Surface

The air-mucus interface is a free, moving surface. The surface tension of the air-mucus interface is in the range of 30–34 mN/m [45–47]. The effect of surface tension is incorporated into the stress balance boundary condition through capillary pressure:

$$\hat{n}\cdot T\cdot \hat{n}=P_{cap},\;\hat{n}\cdot T\cdot \hat{t}=0$$ (6)

$$P_{cap}=\sigma \kappa$$ (7)

where $\hat{n}$ and $\hat{t}$ are the local unit normal and tangential vectors, ***T*** = −*p****I*** + ***τ***, is the total stress tensor, *P*_*cap*_ is the capillary pressure, *σ* is the surface tension, and *κ* is the curvature. In our simulations we use *σ* = 30 mN/m. We note here that we assume a (low) surface tension, which is constant in space and phenomena such a Gibbs-Marangoni effects [49] are not considered in our simulations. To keep track of the free surface, a Marker and Cell (MAC) method [48] is employed. Briefly, the MAC method places “artificial” particles near the free surface. The movement of these particles is dictated by the velocity of the fluid. In order to include the effects of surface tension, the curvature of the free surface is estimated numerically using a cubic spline fitting.

## Results

### Swirling Flow

This study of swirling flow is chosen to validate the algorithm and to compare the behavior of Newtonian and viscoelastic fluids, prior to a study of HBE cell cultures. We examine the swirling flow of 4 different types of fluids:

1. *Newtonian*: Constant viscosity as a function of shear rate and zero normal stresses
2. *Viscoelastic with a single UCM mode*: Constant viscosity as a function of shear rate and nonzero normal stresses
3. *Viscoelastic with a single Giesekus mode*: Viscosity decreases as a function of shear rate and nonzero normal stresses
4. *Viscoelastic 5-mode Giesekus model*: In addition to the nonlinear behavior captured by the Giesekus model, this model captures the effects of more than one mode of relaxation. In mucus, this multimode behavior may arise from the presence of different types of mucin molecules within the gel network.

The parameters used in the following numerical simulations are given in Tables 1–3.

**Table 3:** Simulation parameters. The value used for the angular velocity is the same as the largest angular velocity of the oscillatory driving condition given by Eq (5).

Parameter	Value
Cell culture radius, *R* [m]	1 × 10^−2^
Cell culture height, *Z* [m]	1 × 10^−4^
Initial depth of mucus, *H* [m]	5 × 10^−5^
Solvent viscosity, *η*_*s*_ [Pa∙s]	1 × 10^−3^
Surface tension, *σ* [N/m]	3 × 10^−2^
Polymer viscosity for single mode models, *η*_*p*_ [Pa∙s]	10
Relaxation time for single mode models, *λ* [s]	10
Angular velocity for swirling flow, *ω*_0_ [s^−1^]	1.57 × 10^−2^
Mucus Density, *ρ* [kg/m^3^]	1 × 10^3^

**Fig 5:**
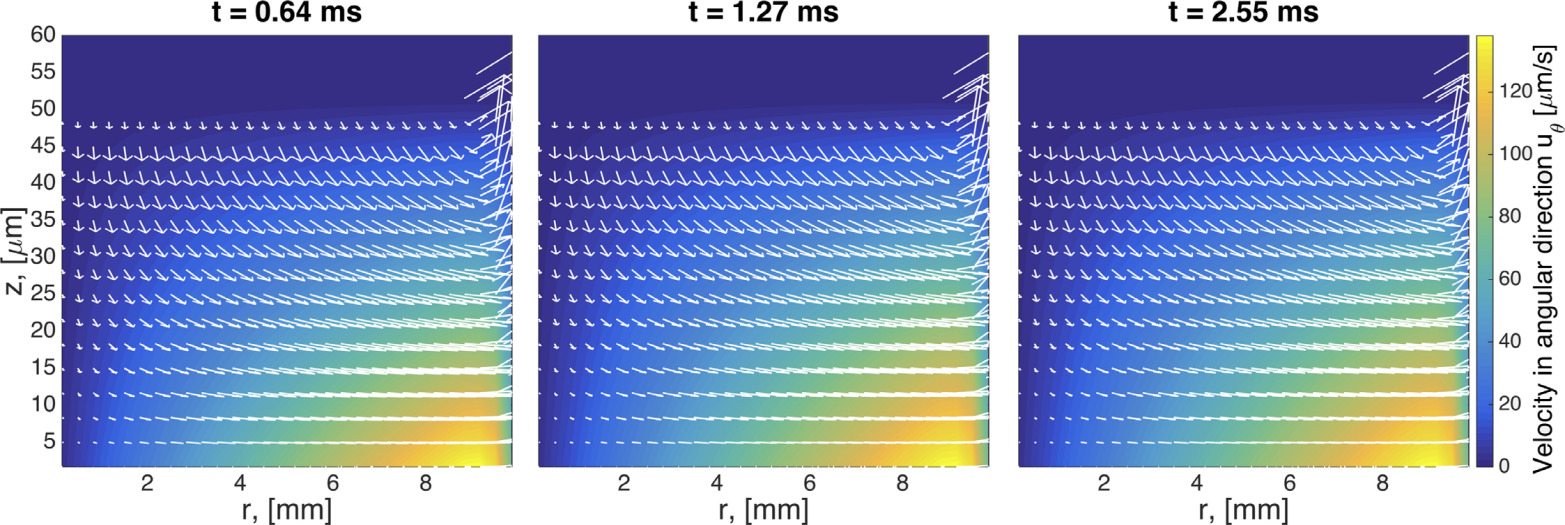
Transient snapshots of the flow field for a Newtonian fluid. The velocity field in the plot is the secondary flow (*u*_*r*_ vs *u*_*z*_) and the color map in the background is the primary flow (*u*_*θ*_). Note that the length scales in these plots differ by one order of magnitude, as *z* is given in microns and *r* in millimeters. Geometric parameters used in this simulation are given in Table 3. The imposed driving condition for swirling flow is *ω*_0_ = 0.0157 Rad/s.

In Fig 5, we show the development of secondary flow patterns at various times at the beginning stages of the swirling flow for a Newtonian fluid. Since *u*_*θ*_ is the primary velocity component for a rotational flow, the secondary flow refers to the (*u*_*r*_, *u*_*z*_) components of the velocity field. At early times an outward centrifugal force causes the fluid near the rotating disk to flow radially outward, up the sidewalls of the cylinder, inward along the top, and finally down near the center. A vortex is formed, and after a certain amount of time, the flow will reach a quasi-steady state.

**Fig 6:**
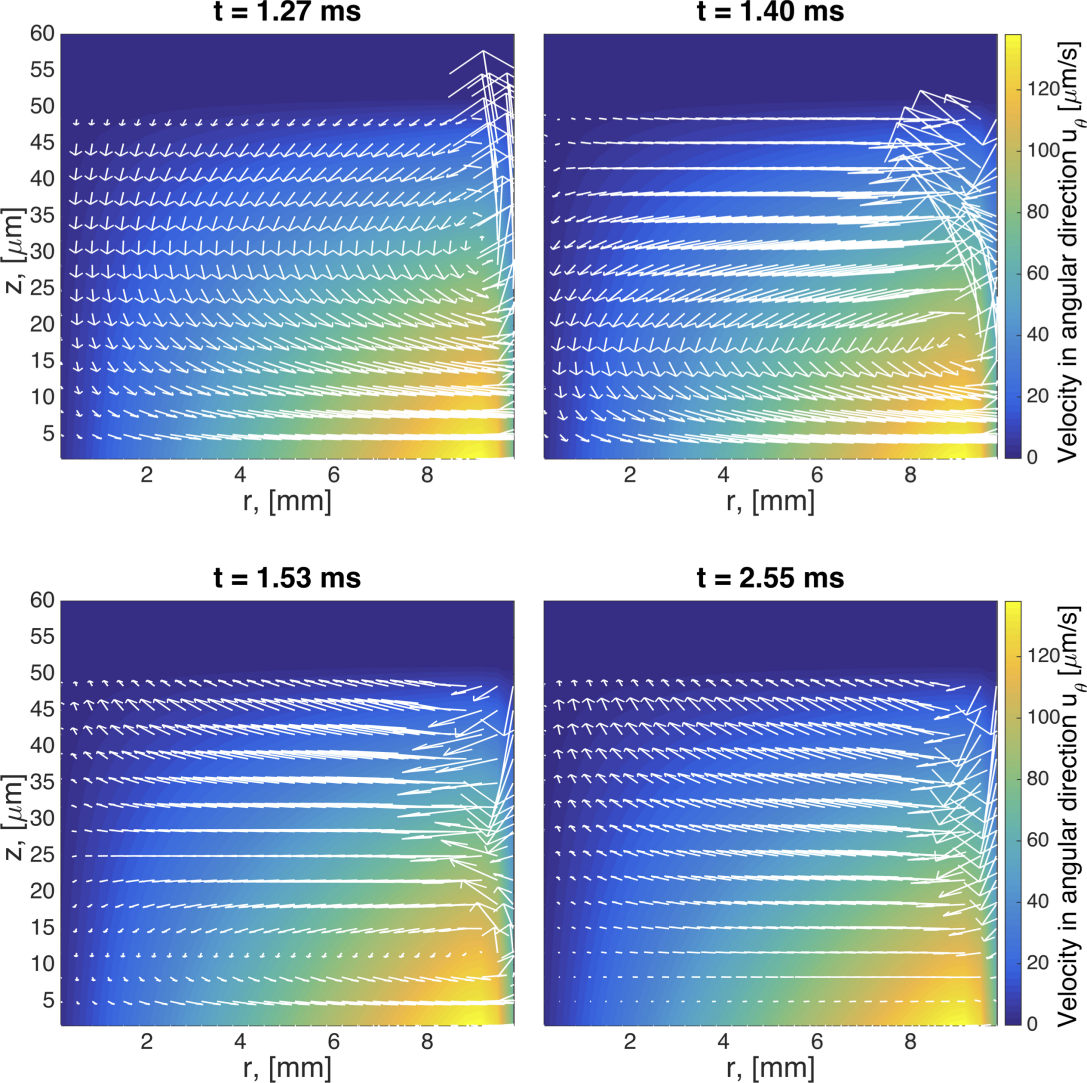
Transient snapshots of the flow field for a single mode UCM model. Times are selected to capture the formation and evolution of a vortex in the upper right hand side of the domain. Geometric and model parameters used in this simulation are given in Table 3. The imposed driving condition for swirling flow is *ω*_0_ = 0.0157 Rad/s.

**Fig 7:**
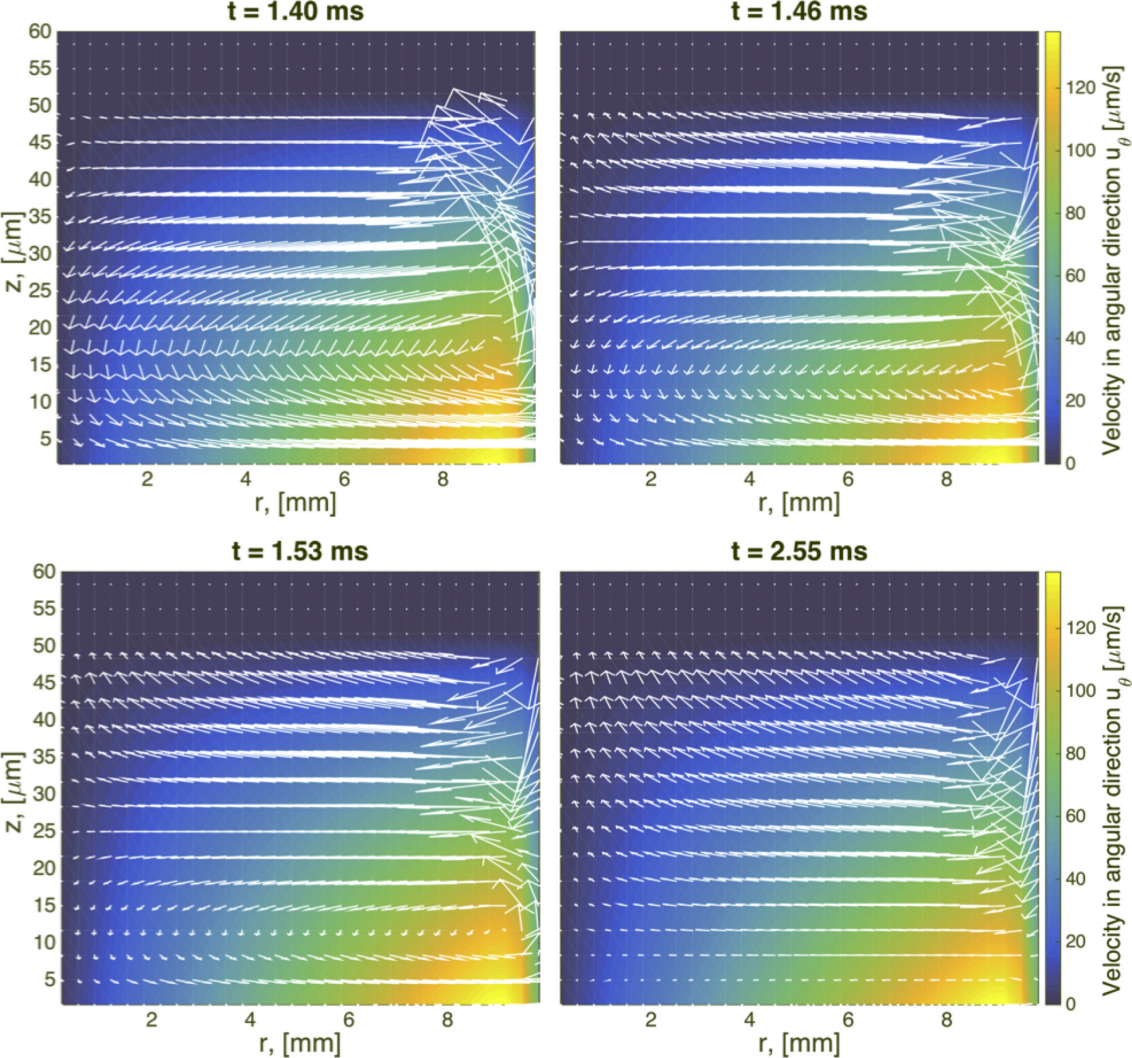
Transient snapshots of the flow field for a single mode Giesekus model with mobility parameter α = 0.3. Geometric and model parameters used in this simulation are given in Table 3. The imposed driving condition for swirling flow is *ω*_0_ = 0.0157 Rad/s.

**Fig 8:**
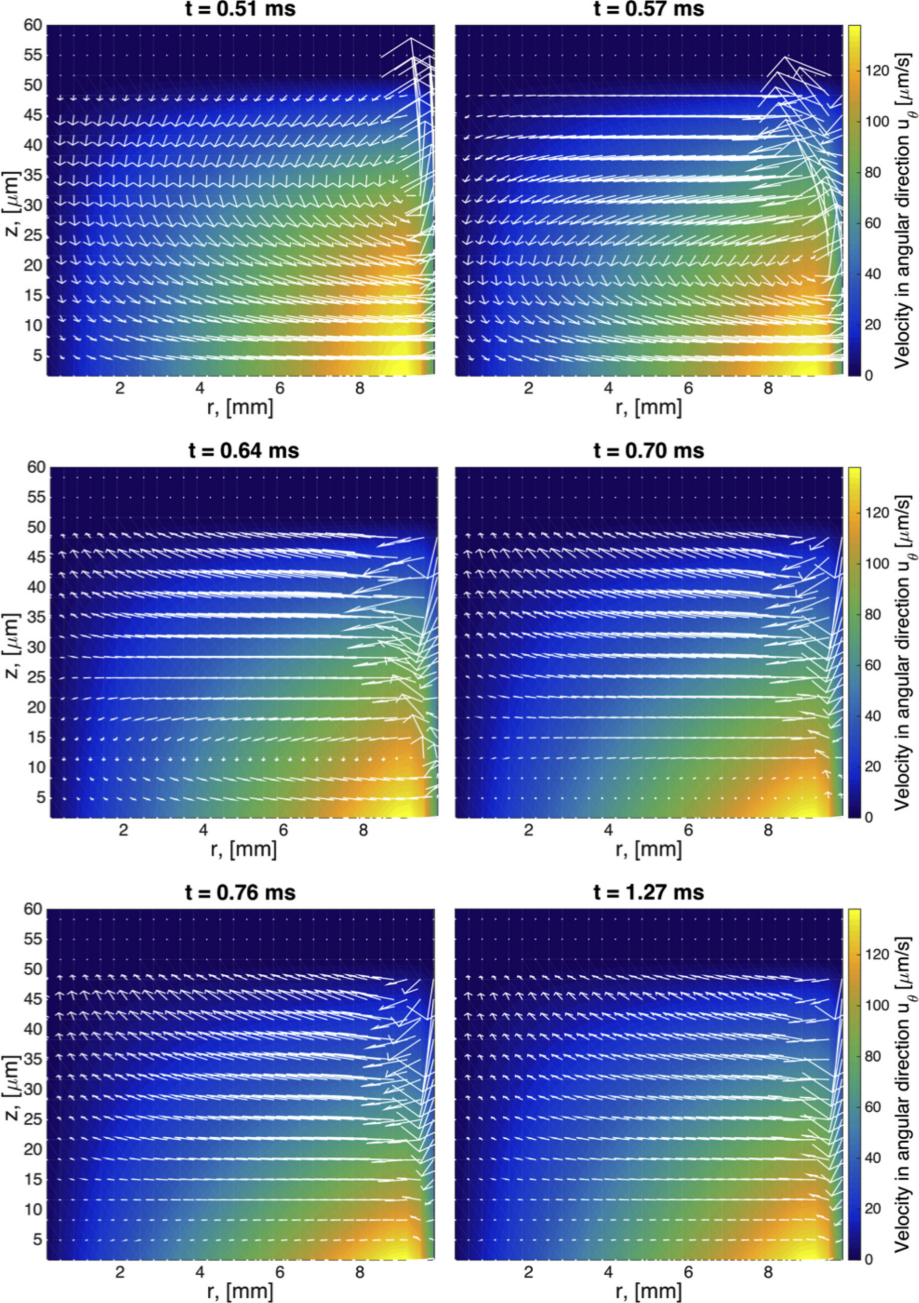
Transient snapshots of the flow field for a 5-mode Giesekus model with parameters specified in Table 1. Geometric and other model parameters used in this simulation are given in Table 3. The imposed driving condition for swirling flow is *ω*_0_ = 0.0157 Rad/s.

**Fig 9:**
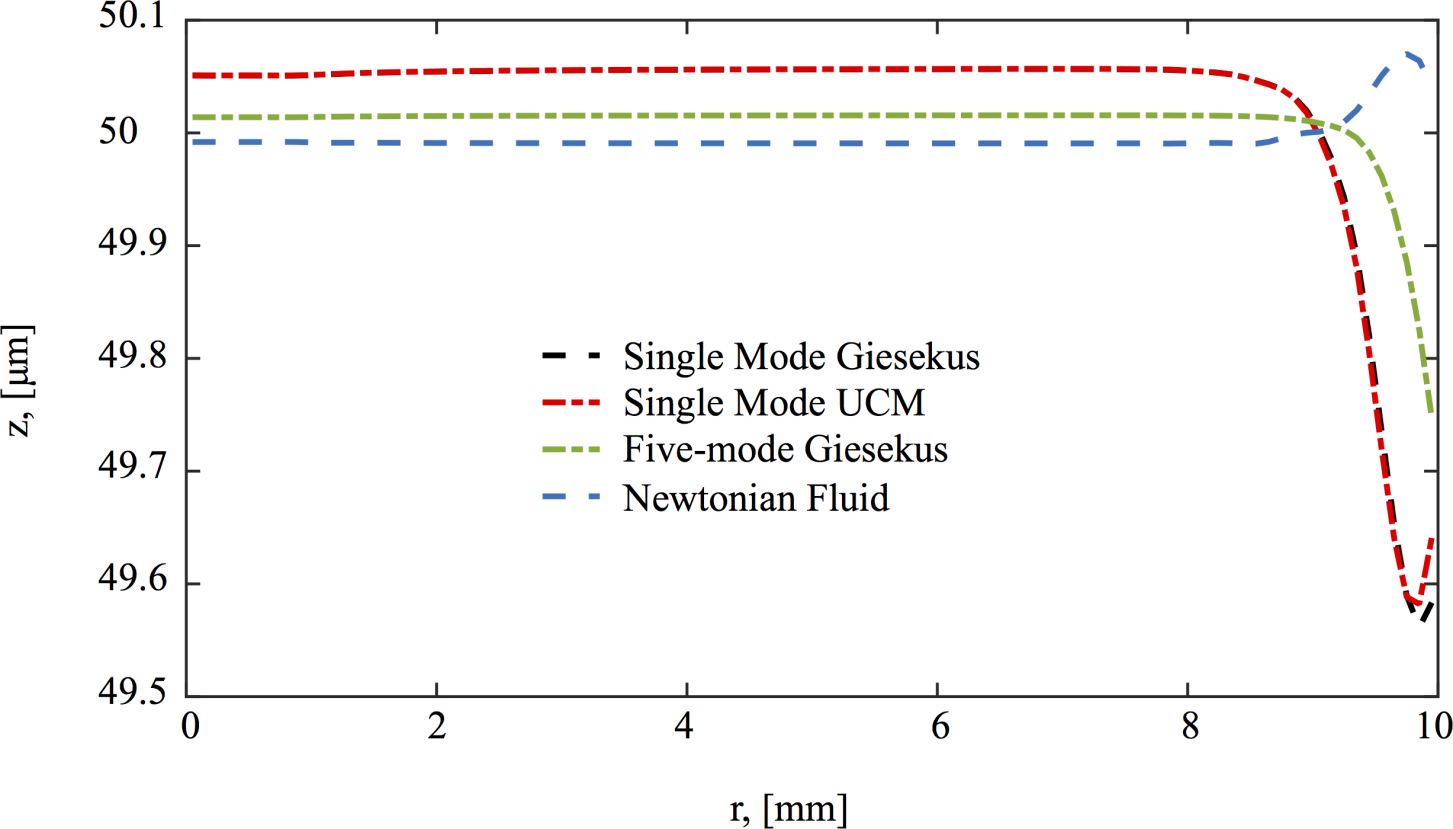
Steady state surface shape is shown for the different models used in this study. Geometric and model parameters used in this simulation are given in Table 3. The imposed driving condition for swirling flow is *ω*_0_ = 0.0157 Rad/s.

The same type of flow is shown for viscoelastic fluids in Figs 6–8. At early times, the secondary flow pattern and vortex are similar to those in the Newtonian case. However, as time evolves, this apparently stationary structure destabilizes, breaks up, and forms a new vortex, which circulates in the opposite direction. This reverse orientation in the secondary flow is a classical consequence of the generation of normal stresses in viscoelastic fluids; a property shared in all our viscoelastic models. In the quasi-steady state of viscoelastic fluids, the fluid near the rotating disk flows radially inward, up the center of the cylinder, inward along the top, and finally down near the sidewalls. In stark contrast, for Newtonian fluids, the flow near the center is downward while upward near the wall. This phenomenon has been long recognized in swirling cylindrical flow, called the Quelleffeck [50], an analog of the Weissenberg effect of rod climbing [50]. The effect that these different behaviors have in the resulting surface shape are shown in Fig 9. While for a Newtonian fluid the flow ‘goes up’ at the walls (just like it does when you stir your coffee), viscoelastic models show a ‘dip’ at the wall. The magnitude of this dip is decreased when multiple relaxation modes are considered.

**Fig 10:**
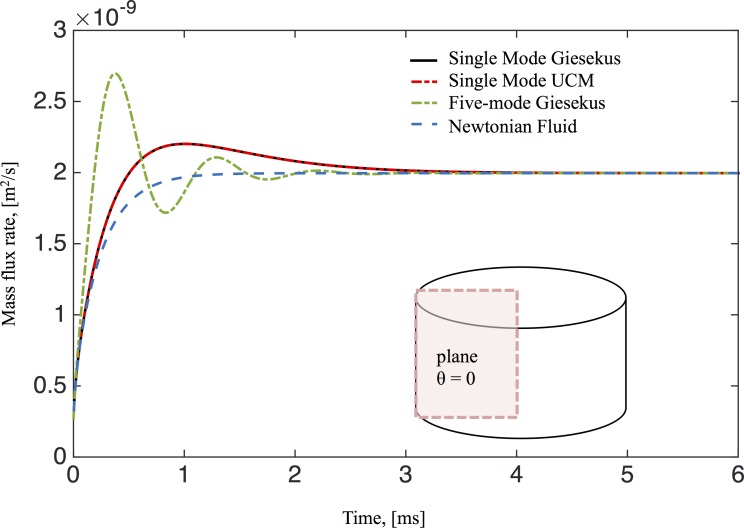
Mass transport flux rate across the θ = 0 plane for the different models used in this study. Calculation of the mass flux is discussed in the text. Parameters in this simulation correspond to those used in Fig 9.

The mass flux rate across the *θ* = 0 plane for all the studied fluids is shown in Fig 10. For Newtonian fluids the mass flux monotonically increases during the transient phase and plateaus in the steady state. However, for single-mode Giesekus and UCM model fluids in the early transient phase, the mass flux rate first increases, similar to the Newtonian flow, then decreases to converge to its steady state. The maximum mass flux rate occurs during the formation of the reverse vortex at the bottom corner of the cell culture. In the transient phase, the 5-mode Giesekus model is non-monotone, reflecting the effects of having more than one relaxation mode. Since each fluid model has finite memory, the stationary mass transport rates are identical, irrespective of the differences in secondary flow patterns. Thus there is no enhancement of mass transport for unidirectional swirling flow at steady state due to viscoelastic effects.

### Cilia-Driven Flow in a Cell Culture

In this section, we discuss numerical simulations of mucus flow in HBE cell cultures using the 5-mode Giesekus model from Table 1 as the constitutive model for mucus. The proxy for the coordinated cilia driving condition is a piece-wise sinusoidal angular velocity function *f*(*t*) at the bottom plate given by Eq (5).

**Fig 11:**
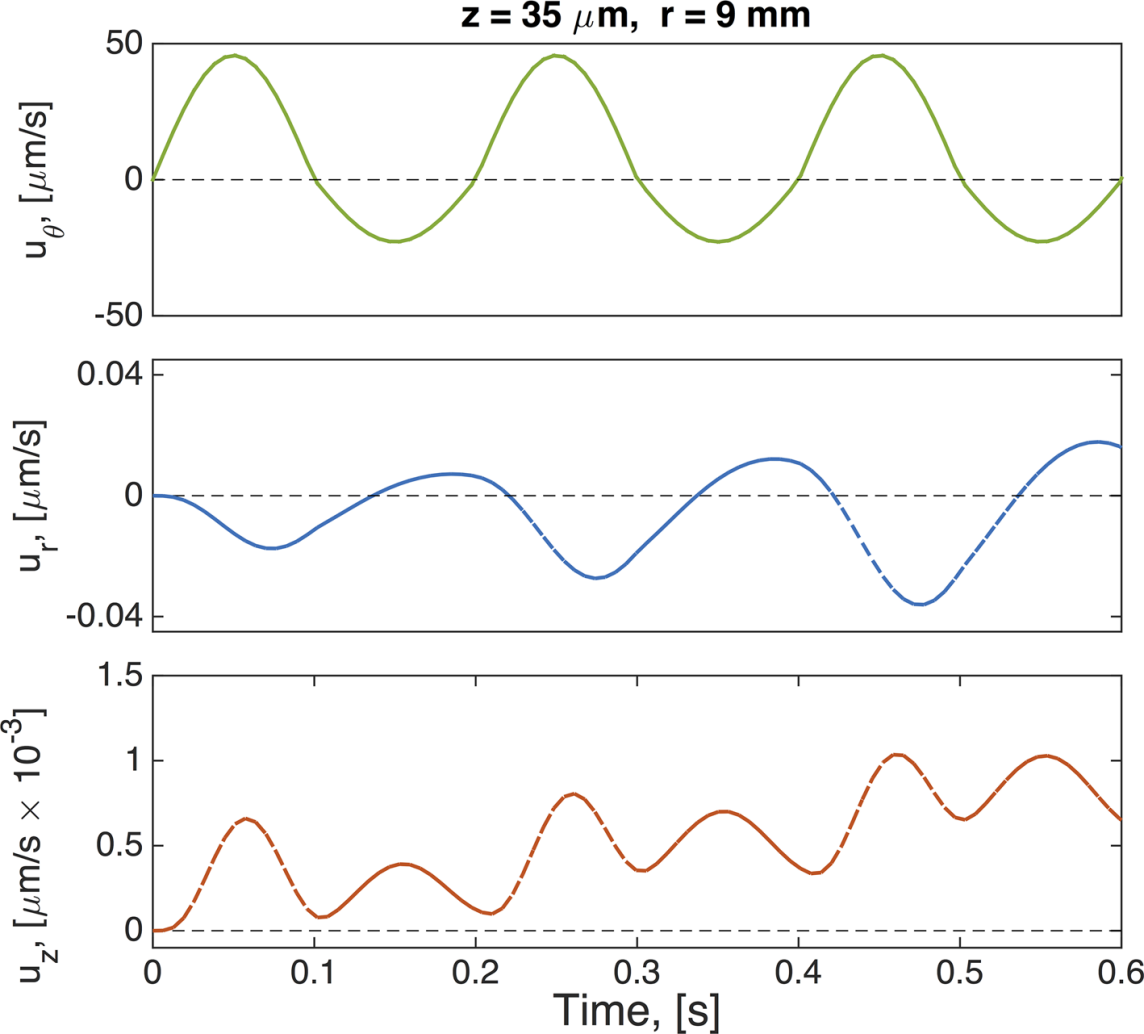
Velocity time series for a 5-mode Giesekus model. Parameters are given in Tables 1–3 and the oscillatory driving condition is given by Eq (5).

**Fig 12:**
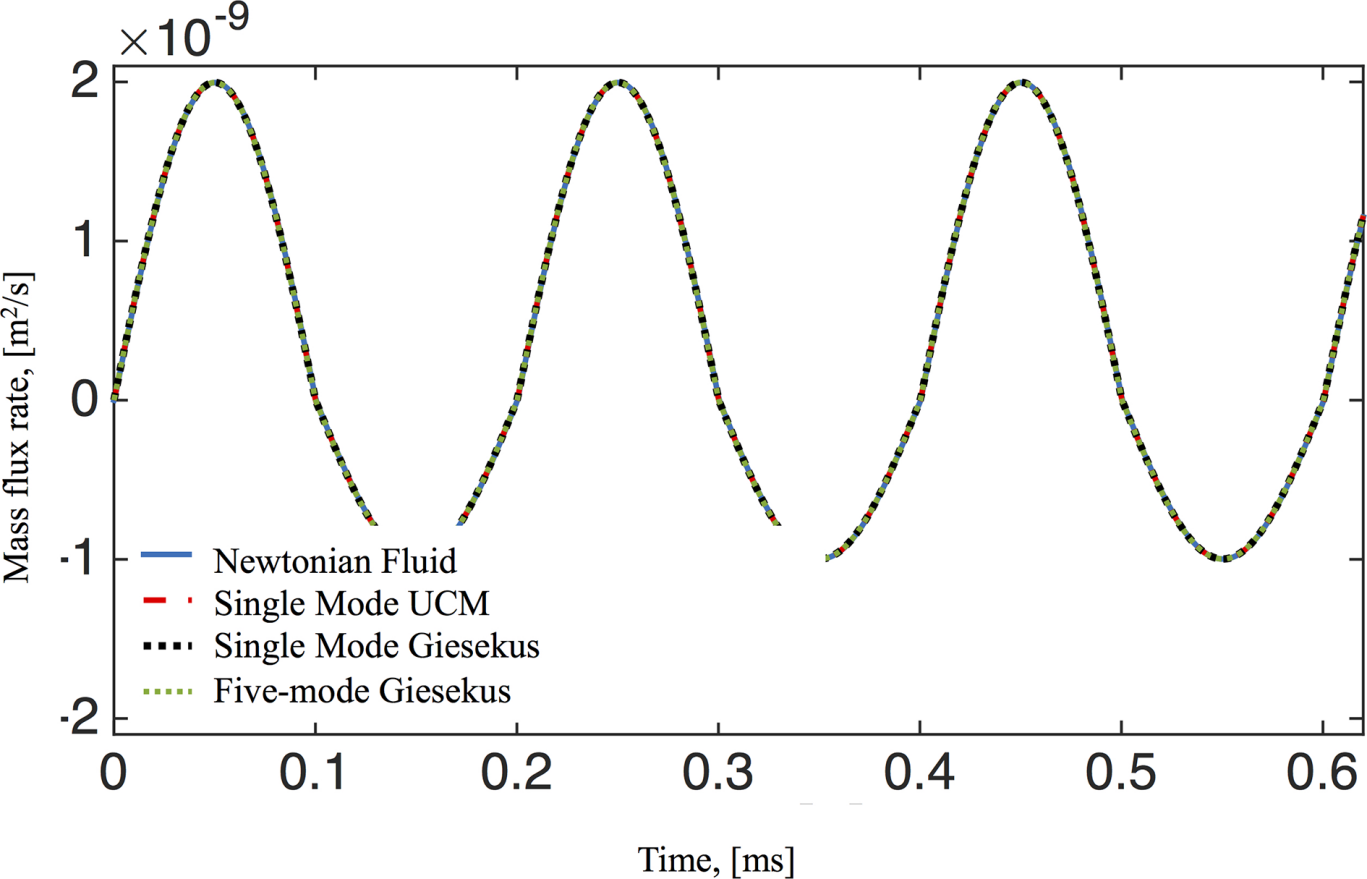
Mass transport flux rate across the θ = 0 plane. The oscillatory driving condition is given by Eq (5) and model parameters are given in Tables 1–3.

Inspection of the secondary flow profiles indicates that as before, the early transient phase matches the viscous fluid structure, which then destabilizes and reverses orientation of the vortex, creating a downward flow near the wall and upward flow at the center of the culture. In Fig 11 we show profiles of velocity versus time for the three components of the velocity field at a point within the vortex that is formed in the upper right side of the computational domain (see for example Figs 5–8). Due to such vortex, the velocity component in the z-direction takes longer to achieve steady state as compared to the other two components. The mass flux rate across the *θ* = 0 plane is shown in Fig 12 for all four constitutive models (viscous, UCM, single mode Giesekus, 5-mode Giesekus), with the result that they all converge to the same bulk flow rate for the pulsatile lower plate driving condition after transient differences.

**Fig 13:**
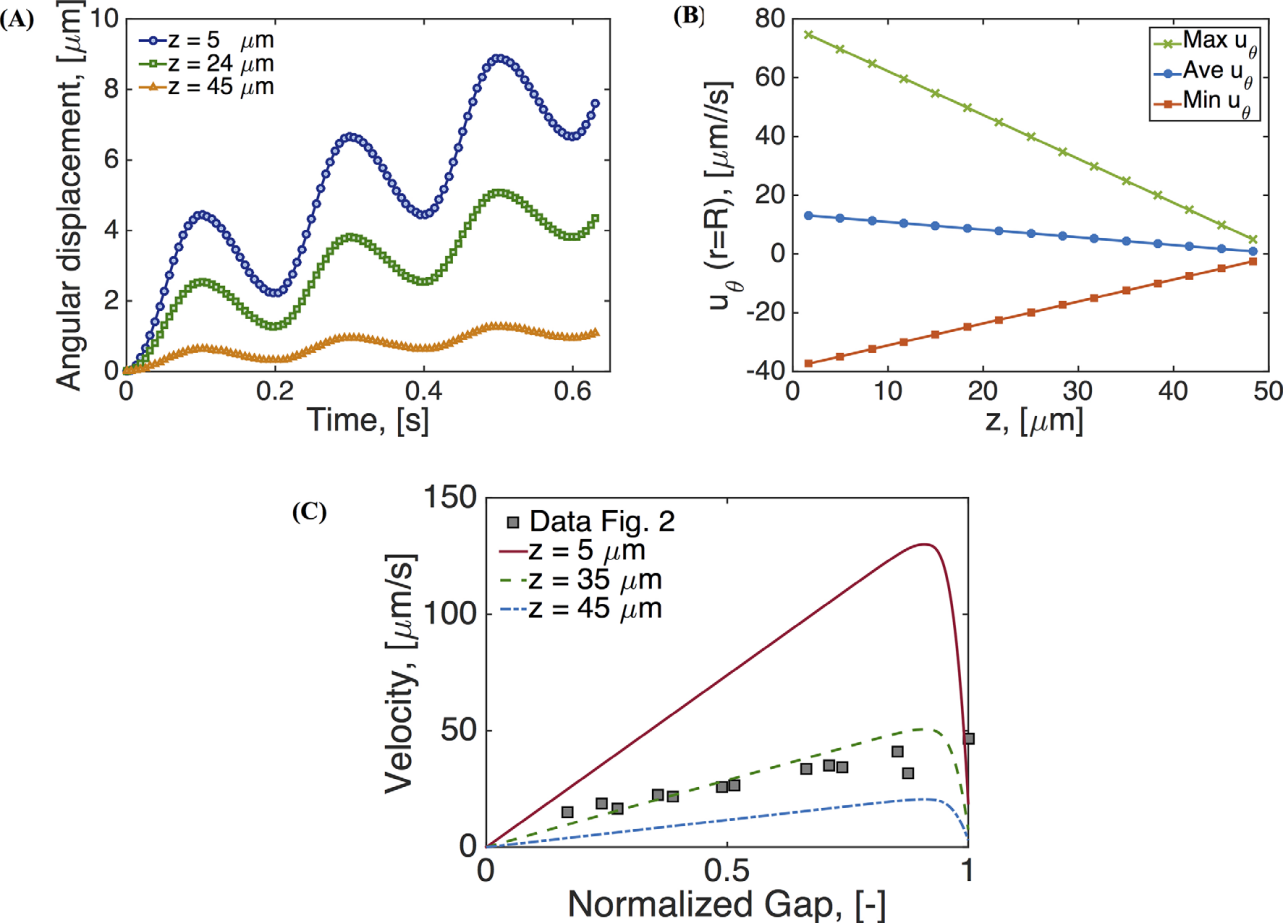
Results for a 5-mode Giesekus model simulation. **(A)** Displacement along the angular direction at the edge of the cell culture at three different heights, **(B)** angular velocity at the edge of the cell culture as a function of height, **(C)** comparison of resulting velocity profiles with those reported in [14]. Parameters are given in Tables 1–3.

Next in Fig 13, we analyze the primary flow field, *u*_*θ*_. Fig 13A shows displacement time series at the edge of the cell culture at different heights. Fig 13B shows that the azimuthal velocity, *u*_*θ*_, exhibits a linear dependence across the gap. It can be shown that the shear strain and shear rate envelopes across the gap are also linear, corresponding to the so-called gap-loading limit that is designed and exploited in rotational rheometers [51, 52]. We note that this gap-loading behavior holds for this current set of parameters, and we return below to explore conditions that induce nonlinear primary flow behavior and departures from the gap-loading limit. Fig 13C compares these linear profiles, predicted by the 5-mode Giesekus model, with measurements from particle tracking velocimetry shown in Fig 2B and adapted from [25]. In that figure, we have normalized the gap in order to plot model predictions and experimental data within the same limits.

### Large Amplitude Oscillatory Shear (LAOS) Analysis

The linear primary flow profiles, shear strain, and shear rate envelopes from the last section imply two things: the flow conditions fall within the gap-loading regime and the driving conditions induce a linear viscoelastic response, i.e., steady state results from Giesekus models overlap with those of the UCM model. However, to rigorously assess the linear or nonlinear regime for the given driving conditions, the standard procedure is to impose a sinusoidal shear strain at the bottom plate, rather than the angular velocity boundary condition in our simulations thus far. To calculate the driving condition for *u*_*θ*_ that is equivalent to an imposed sinusoidal shear strain, *γ*, we consider the following

$$\gamma =\gamma_{0}\sin\left(\omega_{0}t\right)\;\Rightarrow \;\dot{\gamma }=\gamma_{0}\omega_{0}\cos\left(\omega_{0}t\right).$$

In cylindrical coordinates, for an axisymmetric flow, the shear rate is given by

$${\dot{\gamma }}_{r\theta }=\frac{1}{r}\frac{\partial }{\partial r}\left(\frac{u_{\theta }}{r}\right),$$

equating the two equations for the shear rate, gives the following expression for *u*_*θ*_,

$$u_{\theta }=\gamma_{0}\omega_{0}r\cos\left(\omega_{0}t\right)\ln\left(\frac{r}{R}\right).$$ (8)

At the bottom plate we seek to have *u*_*θ*_ / *r* = *P*_0_, where *P*_0_ corresponds to the amplitude of the forward stroke imposed by Eq (5). Choosing *r* = 0.99 *R* to be the point where this happens, gives *γ*_0_ = 0.05. We apply a Fourier decomposition to the resulting shear rate, and shear stress, and checks for higher harmonic generation in the flow field [53–55]. We found that there are no higher harmonic amplitudes greater than 3% of the fundamental harmonic. Furthermore, everywhere in the cell culture, the shear strain is a sine function and the shear rate is a cosine function indicating a linear response.

**Fig 14:**
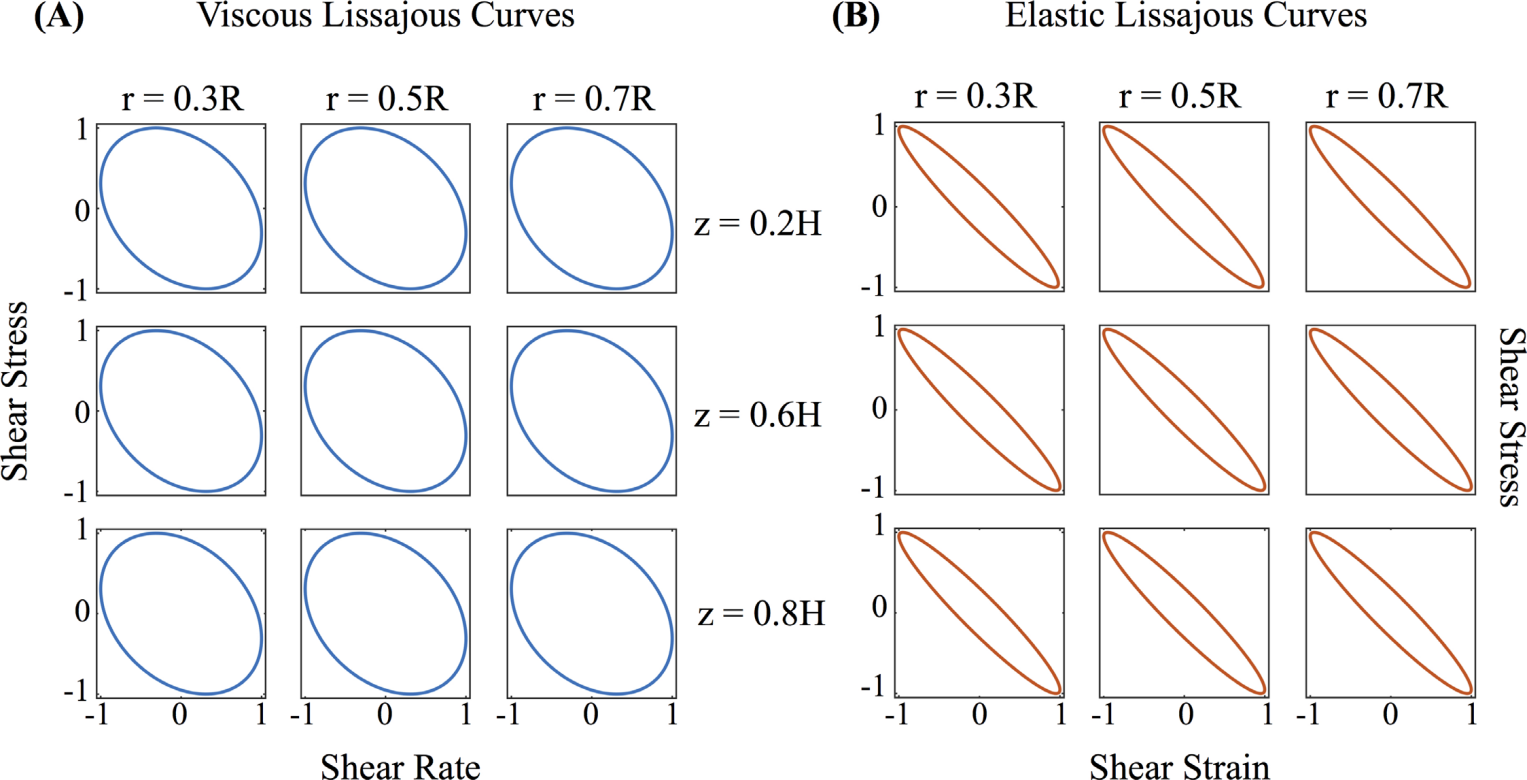
Normalized Lissajous curves. **(A)** shear stress vs shear rate and **(B)** shear stress vs shear strain at different positions of the cell culture.

**Fig 15:**
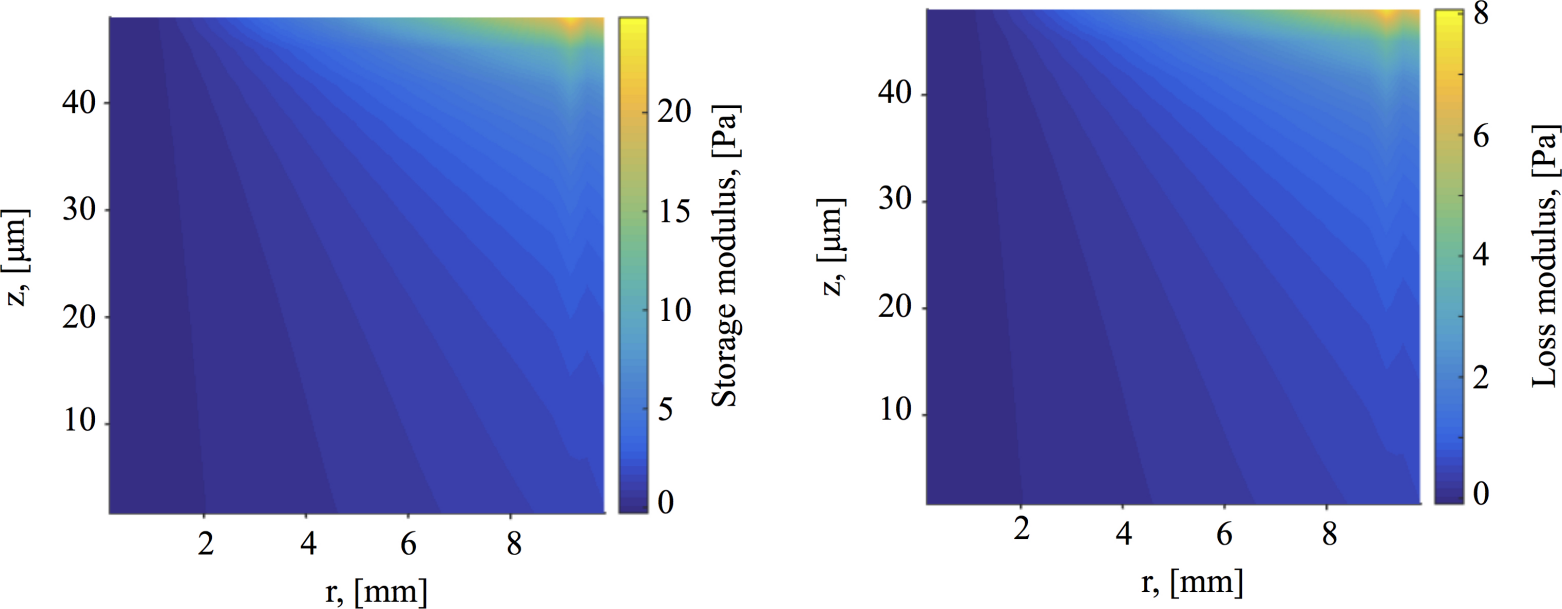
Dynamic moduli. **(A)** Storage modulus *G*′, and **(B)** loss modulus *G*″, everywhere in the cell culture for a 5-mode Giesekus model. Note that since this is considered to be in the linear regime, this is equivalent to a 5-mode UCM model.

Fig 14 shows the normalized Lissajous curves at different locations of our cell culture. Lissajous curves offer a qualitative analysis of the stress waveforms [56]. Elastic Lissajous curves show the oscillatory stress as a parametric function of the strain, whereas viscous Lissajous curves show the stress against the rate of strain. In this way, for an elastic solid, the elastic Lissajous curves are represented by straight lines and viscous Lissajous curves by circles, while the opposite is true for a viscous fluid. In the small amplitude regime for a generic viscoelastic fluid, both elastic and viscous Lissajous curves are ellipses. Any departure from a perfect ellipse signals that the nonlinear LAOS regime has been reached. The results shown in Fig 14 imply that, with the prescribed magnitude of the imposed angular velocity, the solution resides in the gap-loading, linear regime of the viscoelastic model. Since we are in the linear regime, we can characterize the viscoelasticity of the mucus flow by calculating the linear storage modulus, *G*′(*ω*), and loss modulus, *G*″(*ω*), versus position in the culture as shown in Fig 15.

### Aspect Ratio of Mucus Hurricanes

**Fig 16:**
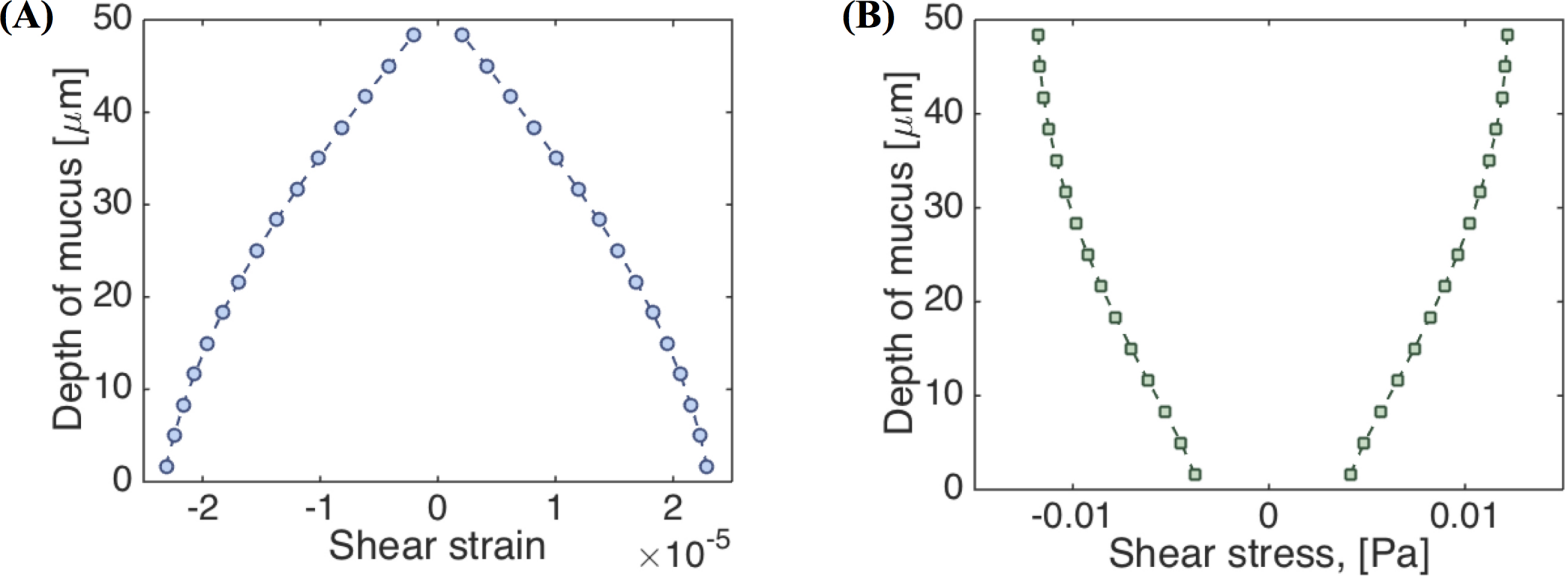
Stress and strain envelopes. **(A)** shear strain and**(B)** shear stress across the gap at *r* = 0.5*R* with *R* = 5 mm. The aspect ratio of the mucus layer is 100, as compared to Fig 15 where the aspect ratio is 200.

**Fig 17:**
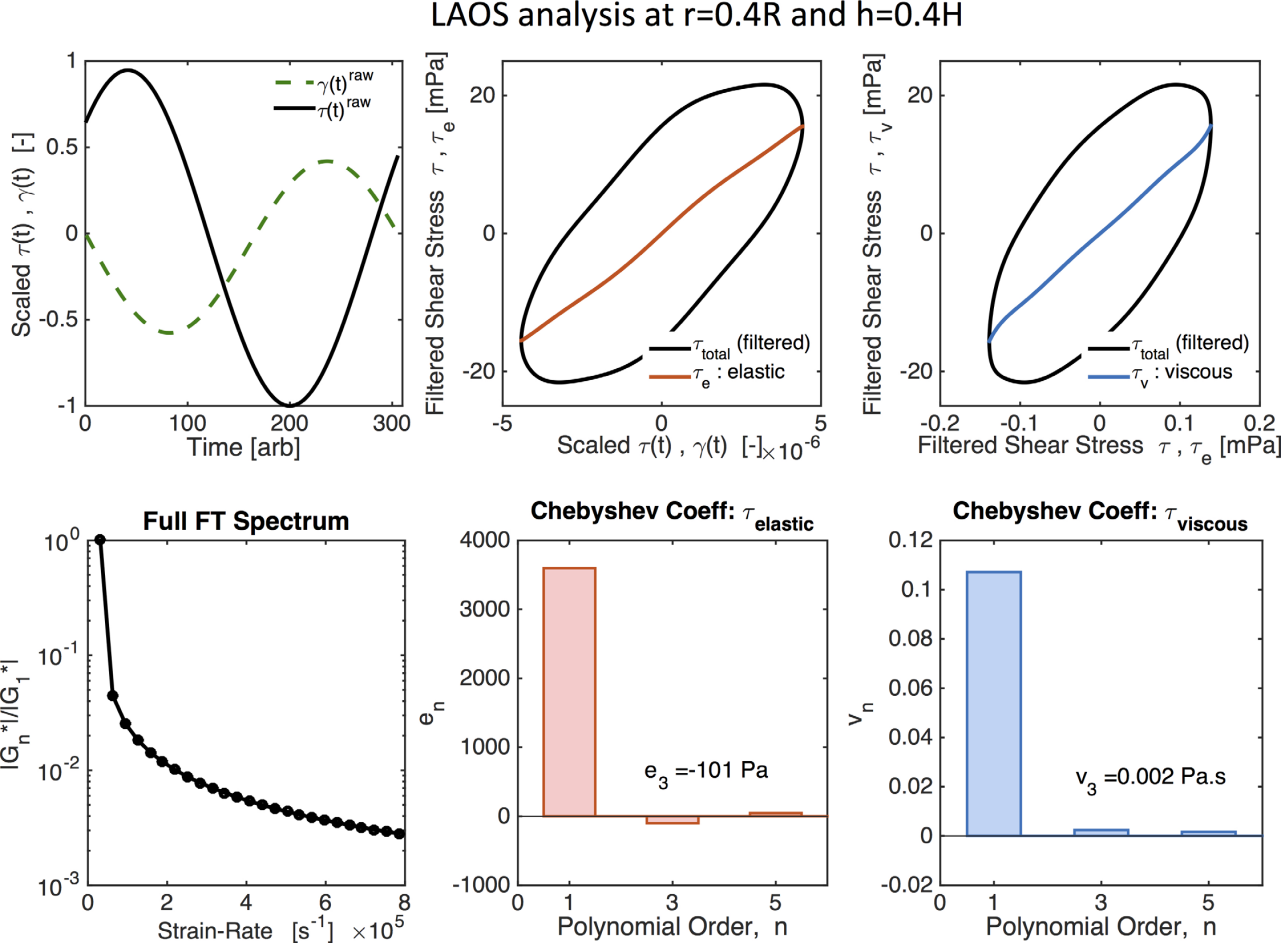
LAOS analysis for nonlinear viscoelasticity. Values are given for *r* = 0.4*R* and *z* = 0.4*H* with *R* = 5 mm. Fig adapted from output of MITlaos software [58].

Next, we explore the implications relative to linear and nonlinear flow when the mucus hurricane is localized in the middle of the cell culture and does not extend all the way to the culture wall. These localized hurricanes are routinely observed among cell cultures. The effective radius of mucus flow is therefore smaller than the cell culture radius R = 10mm. To simulate this phenomenon we assume an effective radius of R = 5mm, outside of which the mucus flow is negligible in comparison and keep the cell height at H = 100 microns. We then choose the magnitude of the oscillatory driving velocity accordingly to match experimental observations. Fig 16 shows the envelopes of shear strain and shear stress across the height of the cell culture at radius r = 0.5R. Since the strain (and strain rate) no longer scale linearly, the shear stress is no longer constant across the gap. Fig 16 indicates that, for these conditions, the system is no longer within the gap-loading limit. The next step is to determine whether the model response is linear or nonlinear. Fig 17 shows LAOS analysis results at *r* = 0.4*R* and *z* = 0.4*H*, where the Lissajous curves now show a nonlinear response. For oscillatory shear with an imposed sinusoidal strain, the resultant stress can be decomposed into elastic and viscous stresses by using symmetry arguments [53]. Ewoldt and coworkers [54] employed an orthogonal decomposition of the elastic and viscous stresses using Chebyshev polynomials, from which signs of the third harmonic Chebyshev coefficients *e*_3_ (elastic stress) and *υ*_3_ (viscous stress) indicate the nature of the elastic and viscous nonlinearities. For our data *e*_3_ < 0 and *υ*_3_ > 0, implying that *the local mucus flow in the culture at this position is strain-softening and shear-thickening*.

**Fig 18:**
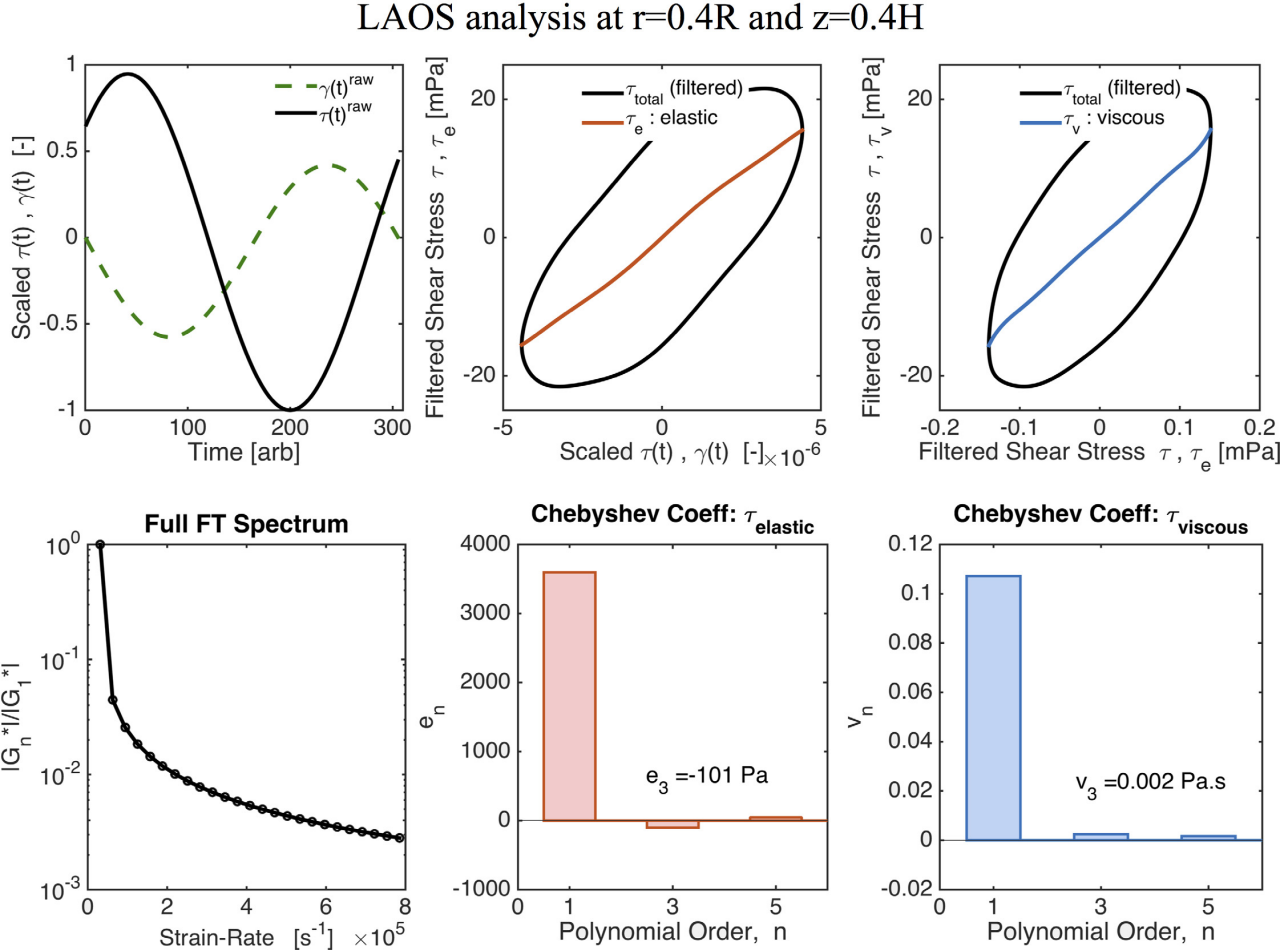
Third harmonic Chebyshev coefficients. **(A)** *e*_3_ (elastic stress) and **(B)** *υ*_3_ (viscous stress) at r = 0.5R and h = 0.5H for different mean driving velocities (*u*_*θ*_) and aspect ratio (R/H).

We now characterize nonlinearity of the mucus flow for reasonable ranges of cell culture parameters with the 5-mode Giesekus mucus model and parameters given in Table 1. Fig 18 shows the third harmonic Chebyshev coefficients as functions of aspect ratio and mean driving velocity. Since the contribution to the elastic stress (*e*_3_) is greater than the contribution to the viscous stress (*v*_3_), we conclude that nonlinearities in the culture arise mainly from to the strain softening of the mucus layer due to the driving force exerted by the cilia carpet. We note here that mucus from different organs and animals may show different behaviors in LAOS. For instance, the work by Vasquez et al [57] found that horse mucus exhibits strain-stiffening.

### Advection-Diffusion of Drug Concentrations

In this section, we consider an initial concentration of drug (or dye) that is dropped at the surface of the mucus layer, which in addition to transport by the underlying velocity field, can also diffuse freely in the HBE cell culture mucus. We assume the drug does not alter the mucus properties or flow field and that there is no affinity between the mucus gel network and the diffusing species. We further assume that the PCL-mucus interface absorbs any drug concentration that reaches it. Finally, we use the flow fields obtained from our previous results using the 5-mode Giesekus model from Table 1 and the cilia driving condition discussed in the Methods section.

For a drug concentration *C*(*t*), with constant diffusion coefficient *D*, the transport of *C* in the mucus layer can be described by the following advection-diffusion equation,

$$\frac{\partial C}{\partial t}=D\Delta C-\nabla ∙(uC).$$ (9)

Here we assume an initial concentration of *C*_0_ and no additional sources. In cylindrical form, the equation becomes

$$\frac{\partial C}{\partial t}=D\left[\frac{1}{r}\frac{\partial }{\partial r}(r\frac{\partial C}{\partial r})+\frac{1}{r^{2}}\frac{\partial^{2}C}{\partial \theta^{2}}+\frac{\partial^{2}C}{\partial z^{2}}\right]-\frac{u_{r}}{r}\frac{\partial }{\partial r}(rC)-\frac{u_{\theta }}{r}\frac{\partial C}{\partial \theta }-u_{z}\frac{\partial C}{\partial z}.$$ (10)

We assume axisymmetric flow, but depending on the symmetry of the initial concentration *C*_0_, the advection-diffusion of *C* inside the cell culture may or may not be axisymmetric. However, if *C*_0_ is axisymmetric, the concentration will remain so. In this case the advection-diffusion equation reduces to

$$\frac{\partial C}{\partial t}=D\left[\frac{1}{r}\frac{\partial }{\partial r}(r\frac{\partial C}{\partial r})+\frac{\partial^{2}C}{\partial z^{2}}\right]-\frac{u_{r}}{r}\frac{\partial }{\partial r}(rC)-u_{z}\frac{\partial C}{\partial z}.$$ (11)

The above equations are solved using a finite difference method.

**Fig 19:**
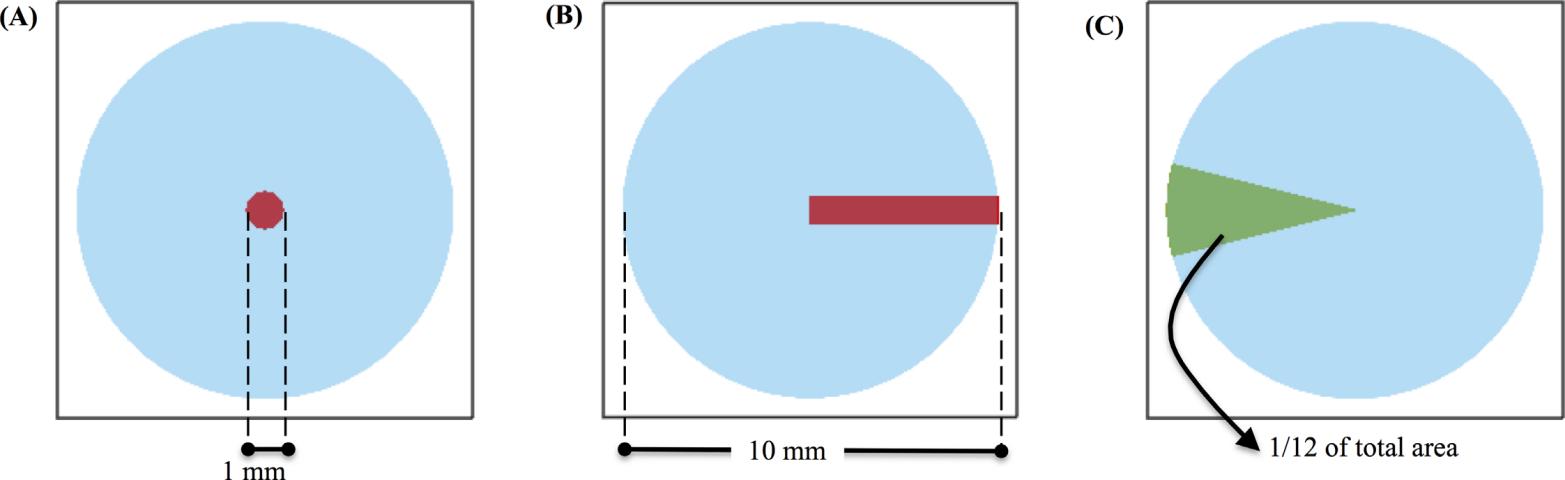
Initial distribution of drug concentration at the air-mucus interface. The distribution is shown in red. **(A)** Disk of radius 1mm and **(B)** strip whose width varying as a function of the radius: 2*πr*/100. **(C)** Area to measure absorption at the bottom plate.

Initially, we set *C*_0_ to be a disk of unit concentration with radius 1 mm at the center of the air-mucus interface. Fig 19A depicts this initial condition with the drug-rich region represented by red. Using this initial condition, we investigate the effect of the diffusion coefficient on the distribution of drug concentration in space and time. The dimensionless Peclet number, *Pe*, describes the relation of advection relative to diffusion,

$$Pe=\frac{advective\;transport\;rate}{diffusive\;transport\;rate}=\frac{L∙V}{D},$$ (12)

where *L* is the characteristic length, which in our case is 1 × 10^−2^ m (radius), *V* is the characteristic flow velocity, which is our case is 1 × 10^−9^ m/s (mean local velocity in the r direction, note the mean local velocity in the *θ* direction is 1 × 10^−4^ m/s) and *D* is varied from 10^−15^, 10^−14^ to 10^−7^ m^2^/s such that the Peclet number ranges between 10^−4^ and 10^4^. We note that the diffusion coefficient for a one-micron particle diffusing in water is ~5 ∙ 10^−13^ m^2^/s.

**Fig 20:**
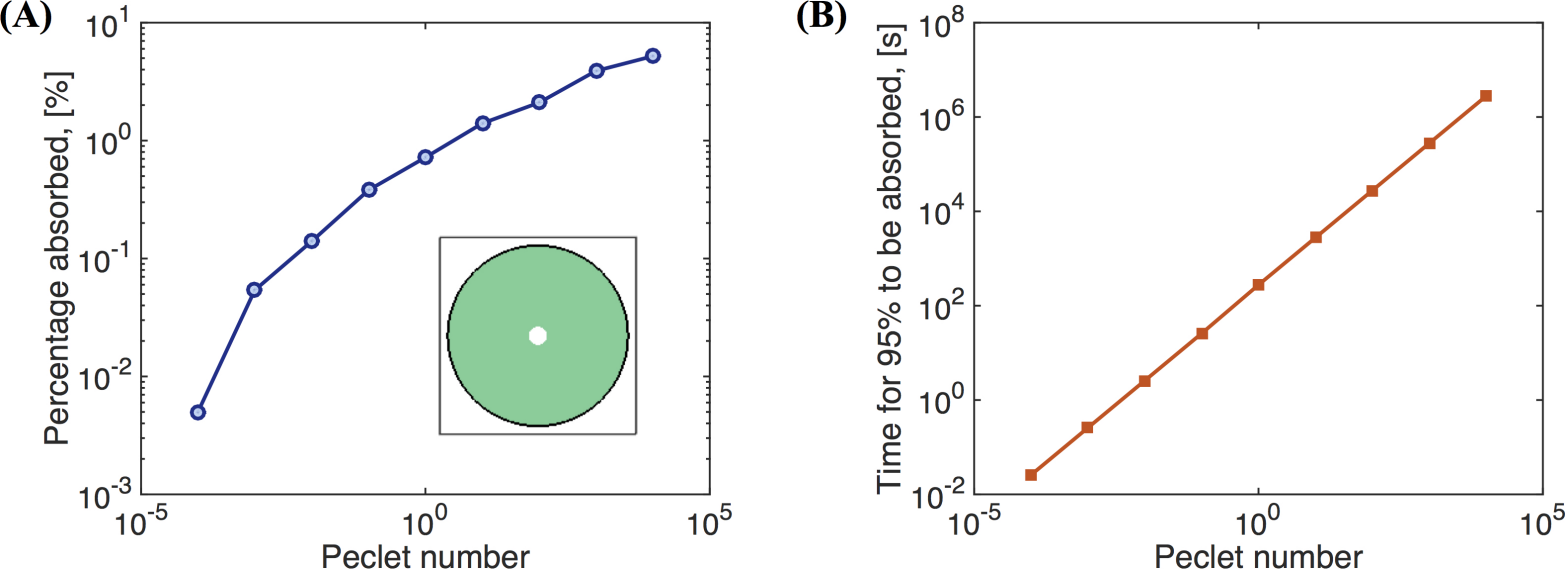
Drug absorption. The percentages of the drug concentration absorbed at the bottom plate on the outer section exterior to the domain of initial concentration (Fig 19A) versus Peclet number. **(B)** The time it takes for 95% of the drug concentration deposited at the surface in a small disk at the center (Fig 19A) to be absorbed on the outer section at the bottom plate versus Peclet number *Pe*.

Fig 20A shows the percentage of the drug concentration absorbed at the bottom plate by the exterior domain to the circle of initial drug concentration (red region in Fig 19A). When *Pe* is small (*D* is large), almost no drug will be absorbed on the outer section of the bottom plate since the drug mainly diffuses in the z-direction due to the large aspect ratio (10 mm radius, 50 μm depth, or aspect ratio of 200). Therefore, with this large aspect ratio and initial condition, most of the drug would be absorbed in a slightly larger radius domain at the bottom plate, with minimal radial diffusion and minimal influence of advection by the mucus flow. Even when *Pe* = 1 × 10^4^ (*D* = 1 × 10^−15^m^2^/s), only 5.2% of the drug concentration is absorbed on the outer section of the bottom plate. Hence the effect of mucus flow on the absorption of the drug concentration is minimal under these conditions. Fig 20B shows the time it takes for 95% of the drug concentration to be absorbed on the outer section of the bottom plate as a function of *Pe*.

**Fig 21:**
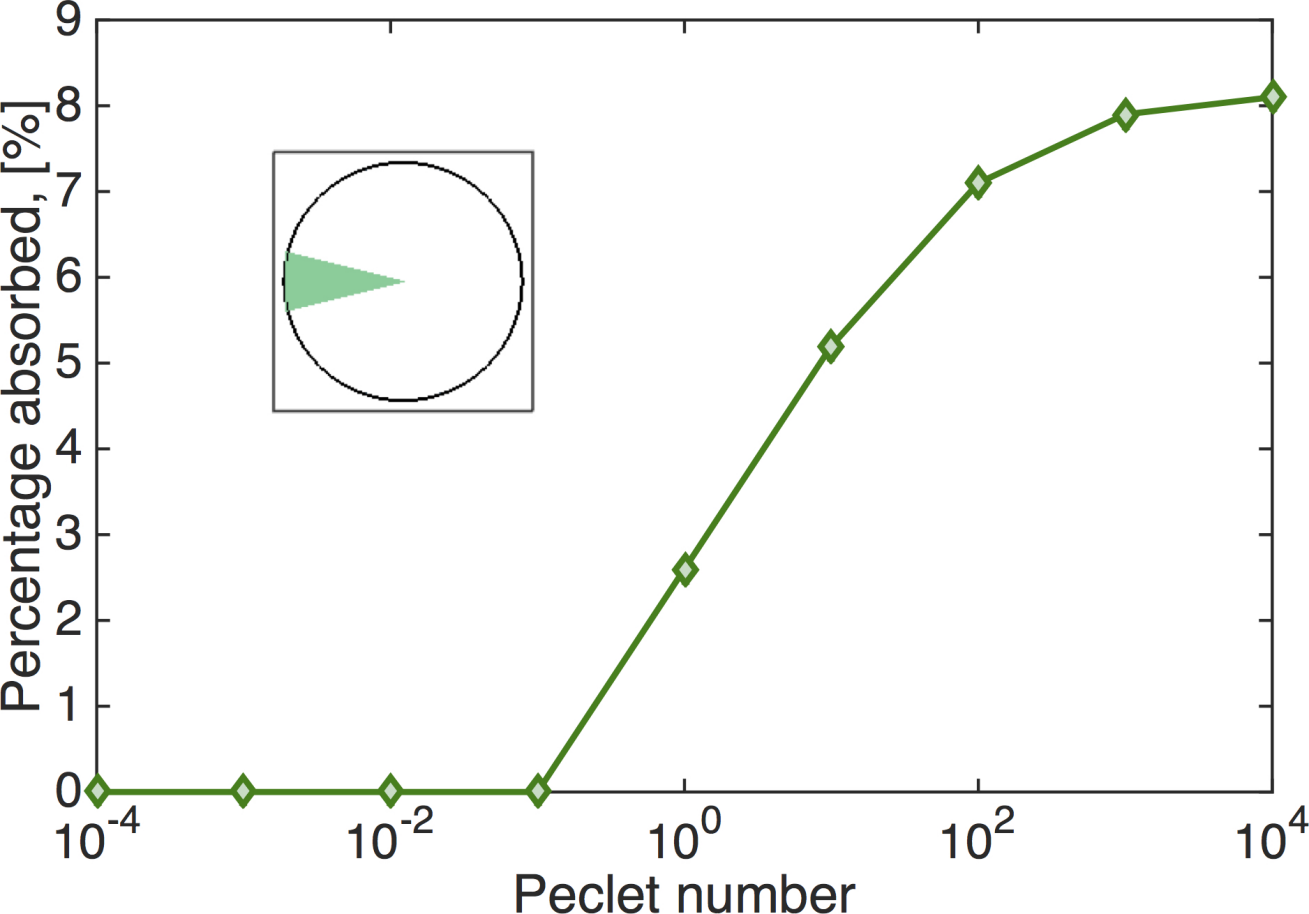
Drug absorption. Percentage of a drug concentration deposited at the surface in the rectangular domain in Fig 19B that is absorbed on the circular sector in Fig 19C at the bottom plate vs. Peclet number.

Next we explore the effects of angular advection on a drug deposited at the air-mucus interface (Fig 19B). To quantify absorption across varying Peclet numbers, we measure the percentage of the drug absorbed on a circular sector at the bottom plate with angular dimensions, −π/12 < θ < π/12 (illustrated in Fig 19C). This area takes up 1/12 (or 8.33%) of the bottom disk and is opposite the initial distribution of the drug. If the drug is evenly absorbed on the bottom plate, this area would absorb 8.33% of the total drug concentration. The closer the percentage absorbed on this area to 8.33%, the greater the influence of advection. Fig 21 shows the result of the percentage of drug concentration absorbed on the circular sector on the bottom plate versus Peclet number. We can see when Pe < 0.1, drug transport is not effective; when Pe > 1, drug transport is effective and when Pe > 10^2^, transport is extremely effective since the percentage is quite close to the "perfect" score of 8.33%.

## Discussion

A multi-mode nonlinear constitutive model for mucus is constructed directly from micro- and macro-rheology experimental data on cell culture mucus, and a numerical algorithm is developed for the culture geometry and idealized cilia driving conditions. These steps toward computational modeling of mucus flow in HBE cell cultures, with an idealized coarse-graining of the cilia driving condition on the mucus layer, yield several meaningful results.

First, the simulations show that viscoelasticity captures the simplest of culture observations: normal stress generation in shear leads to a peak in the air-mucus interface at the middle of the culture and a depression at the walls; furthermore, the corresponding stationary flow profile throughout the culture explains why this dome interfacial shape prevails. A Newtonian secondary flow sets up initially but then destabilizes in the viscoelastic simulations. A reverse-orientation vortex replaces the Newtonian vortex structure in the secondary flow, pushing fluid upward in the center of the culture instead of being pulled downward as with viscous fluids.

Second, model simulations exhibit both linear and nonlinear viscoelastic regimes for the same constitutive model simply by varying the hurricane radius and mean rotational velocity. The linear versus nonlinear characterization refers to viscoelasticity metrics that measure the relative shear thinning/thickening and strain softening/hardening of mucus throughout the cell culture on the basis of higher harmonic generation [53–56]. We predict that mucus hurricanes that occupy the full culture diameter exhibit essentially linear viscoelastic behavior, with negligible higher harmonic generation. In addition, the behavior is consistent with the gap-loading constraints built into shear rheometers. On the other hand, a hurricane, which occupies only half the cell culture radius, can not only violate the gap-loading limit, but also exhibit strain softening and shear thickening within outer bands of the mucus hurricane. This prediction suggests thickness of the mucus layer may determine whether coordinated cilia induce “yield” (softening or hardening, thinning or thickening) in the mucus layer.

Third, we simulate the advection-diffusion of a drug concentration dropped at the surface of the mucus flow versus Peclet number. The Peclet number is tunable in cultures and in the model by modifying mucus (e.g., varying wt% solids), the forcing conditions at the mucus-epithelial interface, or the radius of the mucus hurricane. These simulations explore the uptake profile (at the mucus-epithelial interface) of a small molecule drug dose deposited over some patch at the air-mucus interface, and in particular how the mucus advection profile enhances diffusion and impacts the drug uptake profile. The simulations show that due to the thinness of the mucus layer relative to the radius of the culture, for typical cell culture conditions, a circular patch of drug concentration reaches the epithelium in a slightly wider patch just below the air-mucus interface where it was deposited; i.e., advection has a minimal spreading effect. Thus only a small fraction of the tissue gets the drug dose. We then illustrate how to specify the surface deposition profile that exploits the advection profile to achieve a uniform uptake at the epithelium.

Our modeling-experimental approach fulfills several purposes: to implement linear and nonlinear constitutive modeling of mucus from micro- and macro-rheology; to test constitutive modeling in an independent experimental system; to build a coarse-grained model of the PCL-mucus boundary condition; to measure and understand modifications in mucociliary transport during and after deposition of a controlled drug concentration; to measure and simulate both the flow and stress fields throughout the mucus layer; and to measure and simulate how the advection profiles in the culture couple with diffusion of particulates landing on the air-mucus interface. These results lay the groundwork for extension of the code to three dimensions and more realistic metachronal wave boundary conditions, both in cell cultures and in airways.
